# Leçons Orales de Clinique Chirurgicale, faites à l'Hôtel Dieu de Paris

**Published:** 1834-10-01

**Authors:** 


### Medico-Chirurgical Review,

### No. XLII.

[No. 2 of a Decennial Series.]

JULY 1, to OCTOBER 1, 1834.

### Leçons Orales de Clinique Chirurgicale, faites a l'Hotel Dieu de Paris. Par *M. le Baron Dupuytren*, Chirurgien en Chef. Recueillies et publiées par une Societé de Médecins. Tomes 2 et 3 :—à Paris, 1832, 1833.

[Continued from Page 91, of No. 37.]

It is not, perhaps, necessary to enter into the reasons, which have occasioned the long interval between the former and the present notice of M. Dupuytren's lectures. These lectures have formed a staple article with our contemporaries, at least, with that portion of them whose visits are *not* few and far between; we mean the weekly journals. Yet these journals have presented them at such lengthened intervals, that the chain of connexion and interest is broken, and they resemble scattered and insulated pearls, rather than a close and well-arranged tissue, valuable in its construction as well as its material.

We would recommend all whose education enables them to understand the French language, and whose means permit them to purchase the volumes containing the lectures, not to rest contented with mere translations. We have no hesitation in expressing our opinion, that the Leçons Orales will become a standard work, and will be found in the libraries of all accomplished surgeons. But we cannot acquiesce in the extravagant eulogy which some of our contemporaries have heaped upon it, nor do we feel constrained or inclined to admit, that it throws into the shade the efforts of modern English surgeons.

A candid and a rational consideration will lead, we apprehend, to a more moderate appreciation of its merits. It will probably be perceived to display the peculiar excellencies and defects of the present style of surgical works in France. There will be seen the abundance of all that is deducible from anatomy and pathology—the accurate description—and, in general, the just diagnosis; but there will be also seen the absence of that acquaintance with the powers of remedies, and the want of that decision and judgment in their application, which honourably distinguish the British school of Medicine.

A critical examination will discover, in many instances, another fault—one almost peculiar to the French nation. It is a fickleness and inconstancy in reasoning, as in character—a levity which has marked the disposition of the Gaul from the days when Cæsar first conquered and studied the aboriginal tribes. The French medical or surgical writer of the present day, always talks of a rigorous attention to facts, and inculcates the necessity of absolute induction; yet, in some way or other, it usually happens that his conclusions exhibit the powers of his imagination, rather than the strength and sobriety of his judgment. French works are too often insufferably diffuse, the language and the taste encouraging the "copia fandi." The tired reader struggles through innumerable pages, where the verbiage surrounds him like the tall thick grass of an Indian prairie, concealing the prospect, and almost obscuring the sky.

We cannot be accused of a wish to depreciate continental literature or foreign science. Few have laboured with more zeal or more anxiety to diffuse an acquaintance with both among our brethren. But we trust we are possessed of sufficient patriotism to award justice to our native land, and to claim for her the respect and the praise which are her due. It appears to us ridiculous to assert that English surgeons are below the level of their brethren on the Continent. We would wish to be informed what modern French or German works can be ranked above that of Mr. Brodie on the joints—or can be deemed superior to those of Sir Astley Cooper on fractures and dislocations, and on diseases of the testis? If there be such, we confess that we are unacquainted with them.

The memoirs and the lectures of M. Dupuytren display a smaller share of the defects, and a more preponderating amount of the merits of the French school of surgery, than those of his contemporaries. His mind is cast in the English mould, or, rather, his observation and his vast experience have contributed to diminish that fanciful spirit which distinguishes the great majority of his countrymen. In perusing his observations, we feel that we are holding converse with a practical man, and we do not appear to be regarding the declamatory candidate for the Concours.

Our former notice of the second volume of the Leçons Orales was carried to the last article contained in it—one devoted to the subject of gun-shot wounds.

The third volume of M. Dupuytren's lectures contains eighteen articles—the fourth exhibits fifteen more. The latter forms the completion of the work. We have recommended all who can readily read and translate the French language, to purchase the volumes in their native form. But possibly many are insufficiently acquainted with that language, and probably more can ill afford the sum* to which the work amounts. We think then we shall be doing an acceptable service to our readers, in presenting, in as connected and condensed a manner as our limits and the subjects will permit, an analysis of the two concluding volumes, and of the article on gun-shot wounds in the second.

The émeutes of Paris have given the surgeons of that peaceful capital considerable opportunities of observing the accidents of war. The works of Dr. Hennen and of Mr. Guthrie, familiar as they are to the profession, and embodying as they do the whole of what is known on the injuries of which they treat, will render a copious and particular notice of the Baron's obser-

* The price of the four volumes is one pound, eight shillings, and four-pence.

vations superfluous. We shall limit this portion of our task to selection of what may be novel, curious, or important.

### On Gun-shot Wounds.

This article occupies two hundred pages of the work. M. Dupuytren commences with some general observations on the effects of fire-arms, and remarks that they depend on two principal circumstances:—the nature of the charge, and the distance at which the piece is fired. Without following M. Dupuytren through his remarks on the action of the different varieties of fire-arms, and his exposition of the laws which regulate the course and the arrest of balls, we may glance at one or two particulars.

If a piece is charged with powder but no wadding, the explosion is inconsiderable, yet sufficient to contuse the skin severely, when the charge is received at a short distance. But if wadding is employed, the injury which it produces is determined by the quantum of resistance, and the situation of the body struck. A case of this description occurred to M. Dupuytren. Two individuals quarrelled, and one in the heat of passion discharged his gun, which was loaded with powder only, into the abdomen of the other, who immediately fell dead. The piece had been discharged at the distance of a foot or two. On examining the deceased, the clothes were found torn—the anterior wall of the abdomen penetrated by a hole of more than an inch in diameter—the intestine wounded—and the wadding of the piece in the cavity of the belly.

Suicides frequently forget to load the pistol with ball. The parietes of the mouth are violently distended by the rarefaction of the air. Sometimes the wadding traverses the palatine vault. If the direction of the fire is backwards, the vertebral column offers effectual resistance, but the soft palate is torn, and sometimes the inferior maxillary bone is broken.

Small shot of different dimensions operates in two manners—that is, it strikes *en masse*, or after it has spread. In the former case, its action is more dangerous than that of a single ball, because it lacerates extensively the parts into which it is impelled; but where it has spread, the discharge must, of course, have been remote, and comparatively little mischief is inflicted. Much, however, must depend on the part which is injured. If the eye be struck with a single grain of shot, it is irretrievably lost.

Passing over some pages of familiar remark, we may pause to listen to the observations of the Baron on the action of clothing in modifying the effect of balls, and on the injury produced by what has been termed their *wind*. Articles of dress, especially when woollen, protect in some degree the parts they cover. The hole made by a ball in clothes is always smaller than that in the skin. The ball often enters a limb to a considerable depth, without occasioning any perforation in the dress, which is, consequently, carried before it. In 1814, a French soldier, who was wounded before the walls of Paris, was carried to the Hôtel Dieu. On examining the upper portion of the leg, some pieces of cloth were found buried in the bone. On pulling at these with some force, a kind of wadding was extracted, containing a ball inclosed in a part of the soldier's gaiter. Amongst the wounded received at La Pitié, in July 1830, was a patient who presented a nearly similar occurrence. A ball had entered the abdomen, carrying before it a part of the shirt, by means of which the bullet was extracted; the patient recovered. The difference of size between the wound in the clothing and that in the skin, depends on their respective elasticity. The circumstance should be remembered, as an unacquaintance with it gave rise to the idea that Charles XII. of Sweden was assassinated. The ball had pierced the border of the monarch's hat, and entered the anterior part of the cranium, the opening in which was much larger than that in the hat.

The notion of serious injury from the wind of the ball arises from the difference of elastic resistance in various tissues of the body. If a projectile, nearly spent, or at least much diminished in force, strikes obliquely on a rounded surface, like the thigh, it may pursue its course, without leaving any mark upon the trowser of the wounded man. The limb which has been struck is rendered numb and powerless, and the person falls. On examining the thigh the bone is discovered to be broken, the soft parts disorganized, and the skin apparently uninjured. The fact would seem to be, that the muscles, at the time when the limb is struck, are in a state of tension, and yield to the violence more readily than the elastic skin. If, instead of a limb, the chest should be the stricken part, instantaneous death may be the consequence, and a careful examination may be necessary to disclose its cause.

One ball may inflict several wounds, in consequence of its impinging on some hard substance at first, and being thereby broken into two or more portions. Thus a ball struck the lower part of the spine of the tibia in the right leg, and was divided by it into two pieces. Each, diverging a little, passed through the calf, and lodged in the fleshy part of the left leg, which happened to be placed behind the other.

The observations of the Baron on the lodgment of balls, and on their travelling propensities, are deserving of attention.

When a ball is lodged in any organ or tissue, it either excites inflammation and suppuration, or it does not. If none is occasioned, a cyst is formed, attached on its exterior to the surrounding tissue, and resembling on its internal surface a serous membrane; thus the ball is enclosed in a serous cyst. This is a fact of practical importance, for, in performing an operation for the extraction of a ball, if the cyst is left behind, an accumulation of serum in its cavity ensues, and an encysted tumor is the consequence. In removing the ball, the cyst must either be dissected out, or it must be dressed in the interior with lint, in order to produce suppuration and granulation. If inflammation is excited by the ball, the cyst, instead of secreting serum, gives rise to pus, and ultimately fistulous sinuses are formed, connecting the interior of the cyst with some internal cavity, or with the surface of the body.

When balls travel, their progress may be rapid or slow. If the former, they leave no traces of their passage in the organs which they traverse. If the latter, they are surrounded by the serous apparatus just described. Bodies of all forms may shift their quarters in the body, though such as are narrow and pointed will do so with more ease than those which are spherical and obtuse. M. Dupuytren cautions the surgeon to be satisfied of the situation of the foreign body at the time when he performs the operation. If he cuts in a direction where he may have felt it the preceding day, it is possible that it may have removed in the interim. Balls usually travel from the interior towards the surface, but sometimes they pursue the contrary route.

We may pause for a few moments at the Baron's description of the injuries inflicted by balls upon the bones. Sometimes, merely a contusion is produced; but this is more serious than it seems, for the periosteum is destroyed, or inflames, and necrosis may ensue. That the bones should be fractured in various ways, and shattered in various degrees, can scarcely be productive of surprise. But it might not be anticipated, that the resistance of bone would be sufficient to break or divide a ball. When the latter impinges on an angle of bone, such is occasionally the fact. A ball which struck the spine of the tibia has been separated into two portions, which, traversing the leg, made their exit at separate apertures behind. A nearly similar division has been witnessed, in cases of injury of the flat bones. A Swiss soldier was wounded by a ball, which fractured the right parietal bone, and was divided into two; one portion made its escape through the integuments, whilst the other penetrated the brain, and lodged on the tentorium cerebelli. At the same period, there was in the Hôtel Dieu a patient in whom a ball, having broken the occipital bone, was split into two portions, which still remained slightly united, and were arrested at the opening, over which they were placed, as it were, astraddle.

M. Dupuytren relates a case, in order to shew how difficult it sometimes is to determine if a ball has entirely escaped, or has partially lodged. A Parisian was wounded by a ball, which penetrated above the clavicle, and made its exit behind, near the inferior angle of the scapula. The ball presented no other peculiarity than that of being rather flattened on one facet. The patient appeared to be cured, but, on examining him some time afterwards, a hard substance was felt near the posterior wound. An incision was made on it, and a portion of ball was removed. On weighing this, with what seemed the entire ball, the compound weight was found to be that of an ordinary bullet.

M. Dupuytren relates some cases, to exhibit the extraordinary course of balls. So many have been recorded by Dr. Hennen, Mr. Guthrie, and other surgical writers, that we do not think it necessary to pursue the subject.

The Baron has little to say, or at least he says but little, on that serious affection denominated, perhaps not quite correctly, hospital gangrene. He states that in 1814, when the Hôtel Dieu was extremely crowded, many cases of this description occurred; but that, in 1830, when the wards were not too full, and the arrangements were improved, few instances were met with. Contused wounds are those which are most frequently affected with this species of gangrene, and, in 1830, the hands and the feet were the parts in which it commonly appeared.

The compound fractures produced by gun-shot are usually dangerous and severe. We perceive no novelty in M. Dupuytren's description of the immediate consequences of injuries of this description. We may cite one remark, on the period at which secondary hæmorrhage, from the pressure of a fragment of bone, is observed. It is usually late, and subsequent to that at which bleeding from the separation of sloughs is noticed. M. Pelletan saw a case, in which the hæmorrhage occurred on the 70th day.

We may rest for a moment to listen to the lecturer's description of the different conditions which fragments or splinters of fractured bones may exhibit in a limb. He arranges them methodically into primary, secondary and tertiary splinters. The primary splinters are those which result from the operation of the wounding agent. When left to themselves, they die, and operate like foreign bodies, giving rise to extensive suppuration, or to troublesome sinuses. The second order of splinters are such as preserve some adhesions to the parts with which the bones are naturally connected. These adhesions may be insufficient to preserve their vitality, when they fall into the condition of the primary splinters; or the life of the fragments may continue, and they then contribute to the formation of the callus. The third kind of splinters are such as result from the process of necrosis. They remain in their place till that process is so far completed, as to cause or to facilitate their expulsion. The broken ends of the bone are not, in the mean time, united; but consolidation is going on around in the periosteum, the cellular texture, and the other soft parts, and a bony clasp or ferule is formed, supporting, and so far joining, the fractured bone. If the splinters are too large to be expelled, they are locked up, like sequestra, in this new bone or callus, and fistulous sinuses connect them with the exterior, as in ordinary cases of necrosis. To remove them, it is necessary to apply the trephine, and perforate the fresh bony matter.*

M. Dupuytren observes that, amongst the severe consequences or complications of gun-shot wounds, hæmorrhage, especially of a secondary character, tetanus, collapse, erysipelas, diffuse phlegmon, and inflammations of internal organs, must occupy the foremost rank.

Few remarks on those subjects will be necessary, the attention of the profession in this country having either been lately or sufficiently attracted to them. We may notice the Baron's observations upon hæmorrhage.

It is known that gun-shot, like all contused, wounds are little disposed to bleed in the first instance. Patients, however, occasionally die from immediate hæmorrhage, an example of which, in a case of gun-shot wound of the femoral artery, is related by the Baron. Secondary hæmorrhage is much more common, and occurs under two conditions of the artery—when it has been totally divided, or when it has been only partially wounded. In the first case, the eschar produced by the immediate action of the ball, and the coagulum which forms at the orifice of the vessel, seal and prevent the occurrence of hæmorrhage. Accidental causes may increase the circulation, when the clot and the eschar may prove insufficient to prevent the flow of blood. But, in other instances, the bleeding is delayed till the process of suppuration has separated the sloughs, which is usually from the tenth to the twentieth day. Hæmorrhage, in these cases, is seldom preceded by any distinctive circumstances or signs, excepting, perhaps, a sero-sanguinolent discharge, which sometimes issues from the wound. In primary hæmorrhage, the vessel must be tied at the spot where it bleeds; in secondary hæmorrhage, it must be secured, in sound parts, between this and the heart.

M. Dupuytren has seen secondary hæmorrhage from vessels of small, as

---

* For the reader to appreciate, to the full extent, the preceding observations and description, it is necessary that he should be familiar with M. Dupuytren's description of the process by which fractured bones are united; and with the value of the application of the trephine in cases of necrosis.—EDS.

well as large calibre. An officer in the wards of the Hôtel Dieu had such hæmorrhage from the wound of a branch of the temporal artery. Compression proving ineffectual, M. Dupuytren cut down, and tied the vessel on a roll of diachylon.

Varicose aneurism, or rather aneurismal varyx, is sometimes a result of gun-shot wounds.* M. Dupuytren has witnessed an instance of this sort in the shoulder—and in the case of the subclavian artery and vein. He has also observed it follow the reception of a charge of small shot. An appraiser who was wounded in this manner in the hypogastrium, thighs, and genital organs, discovered, at the end of five or six months, a whizzing in the thigh. This could be distinctly heard on the application of the ear, and resembled that produced by the passage of the blood from a large artery into a vein. When pressure was made above the part, it ceased—when applied below, it was increased. A bandage was constructed, and the patient has since enjoyed good health. In his remarks upon the treatment, M. Dupuytren would seem to recommend a practice, which most practical surgeons in this country tacitly or openly condemn. He observes that pressure is always inconvenient, generally insupportable, and seldom advantageous. Ligature of the artery, above the disease, may answer in a recent, but is always unsuccessful in an old case. He, therefore, advises that a ligature should be placed upon the vessel above and below the seat of the disease. The practice in this country is to do but little. If moderate compression can be borne without difficulty, it is reasonable to employ it. If it cannot be supported, and the complaint is not productive of excessive inconvenience, it is not worth the while of the surgeon to perform, nor of the patient to submit to, any operation. But, if the affection should prove troublesome or dangerous, we suppose that the advice of M. Dupuytren may be taken, and a ligature may be applied upon the artery, above and below the communication with the vein.

We may cast a furtive glance at an interesting subject approached by M. Dupuytren—it is the influence of mental emotions, or of moral agencies, upon the wounded. It has long been remarked that, after a battle, the wounds of the victors have usually done better than those of the vanquished. In civil conflicts, the fact is still more striking. During "the three days," the mortality amongst the triumphant citizens was comparatively much less than that of the beaten military. The reverse was observed in the unfortunate émeutes of June, 1832. The young soldier generally suffers more severely than the veteran—his friends, his home, his family crowd upon his mind, and, under the anguish or the terror of his wound, he bids an eternal farewell to all. The veteran, on the other hand, is more affected and depressed by the loss of a battle or campaign. The effects of sudden and passing emotions are equally conspicuous. A young man, who was doing well with a fracture of the leg, was informed one evening by his landlord, that, unless his rent was paid, his goods must be seized and sold. On the next day, he was found labouring under severe fever and great nervous excite-

* The varicose aneurism is that condition, in which an aneurismal sac intervenes between the artery and vein, with both of which it communicates by an opening in either. The aneurismal varyx is a communication between the artery and vein, without any intermediate sac.—Eds.

ment. A workman, slightly wounded in the hand, died rapidly with nervous symptoms, in consequence of the impression produced upon his mind, by his leaving unprovided a girl he was about to marry. Several of the wounded, in 1832, died in a few hours, on being told that, after their recovery, they would be tried by a court-martial. M. Dupuytren denounces, in terms of strong but just indignation, the atrocious improvement in tyranny adopted by the Government of the barricades. That Government was not ashamed to resort to a barbarous meanness, which the lofty and more noble despotisms of the reign of terror, the consulate, the empire, and the restoration had never condescended to imagine.* Louis Philippe surrounded the hospitals with a military force, and, as the medical public are aware, actually dared to command the surgeons to become the official delators of their patients. That they refused, is no more than might be expected from a liberal and humane profession. The terror inspired by the proceeding and the attempt, had the melancholy effect, which no doubt was intended, of destroying many of the unhappy sufferers.

The Baron next describes the effects of gun-shot wounds on particular portions of the body. He commences with wounds of the head. We are almost induced to notice his remarks on erysipelas, and on diffuse phlegmon of the scalp. The latter is really diffuse inflammation of the cellular membrane beneath the aponeurosis of the occipito-frontalis. It is common after scalp-wounds of every description—frequently mistaken by practitioners for erysipelas—and, when mistaken, fatal. The safety of the patient depends on the employment of incisions.† We believe we must " move on," and we scarcely dare halt at the distinctive symptoms of concussion and compression. Yet they are brief, and may be noticed.

In concussion of the brain, says M. Dupuytren, the patient lies tranquilly —the face is pale—the lids are closed by the depression of the upper—the pupils are much dilated—the respiration so subdued as barely to indicate existence—the beat of the heart and of the pulse scarcely felt.

In compression, the patient is usually restless—the face is purplish—the pupil is contracted—the breathing difficult, the chest being seemingly loaded with mucus—there is stertor, and the pulse is frequent, full, and hard.

We cannot entirely agree with the Baron in this enumeration of respective symptoms. We have not found, and we do not believe, that the pupil is dilated in cases of concussion, and contracted in those of compression; the varieties are too great to warrant the positive antithesis. Neither is the face always turgid or reddened in compression; we have seen it absolutely pallid. The pulse, in the latter, is not always full, frequent, and hard; on the contrary, it is not uncommonly labouring and slow. An allusion to partial or general paralysis might usefully have entered into the description of compression.

M. Dupuytren offers some lengthened remarks on the subject of contusion of the brain. The majority of surgeons and of surgical writers have considered only concussion, compression, and subsequent inflammation. In des-

* " Dont la pensée meme n'etait jamais venue," &c.

† In a report from St. George's Hospital, published in this Journal some three or four years ago, the subject was illustrated by several cases.

cribing contusion, M. Dupuytren has attempted, though without great success, to discriminate the symptoms that distinguish concussion and contusion. We are anxious not to dedicate much space to the lectures upon gun-shot wounds. We, therefore, waive the consideration of this question, and merely stop to notice a case related by the lecturer, in illustration of the following remark:—That if contusion affects only one lobe of the brain, three or four days in general elapse before unpleasant symptoms occur.

*Case.* A young man, 15 years of age, received a violent blow upon the head, which produced a wound, and loss of substance in the bones. For three days he was able to get up, and to walk freely in the ward. On the fourth day symptoms of contusion supervened, and in three days more the patient died. Half of the brain was found disorganized.

We could wish that the Baron had been more explicit in describing the mode of disorganization. At present, we are ignorant if the alteration was such as a blow might have occasioned, or rather the effect of inflammatory action. By the way, we may express a hope that it is not the common practice in the Hôtel Dieu, to allow a patient with a wound of the scalp, and a loss of bone, to walk about the wards immediately after the reception of the injury. If such is the method of treatment adopted, we should think that the Baron must have many opportunities of investigating the morbid changes produced by inflammation of the brain.

Wounds of the face are, in general, not productive of fatal consequences. Yet, occasionally, the secondary effects are very serious. One of the wounded of July had both jaws almost entirely carried away by case-shot. He sank under cerebral symptoms. Many citizens had the lower jaw broken by balls; some were cured—others died from repeated hæmorrhages—and others from inflammation, established in the brain or in the chest. An unfortunate effect of these wounds is the profuse and fetid suppuration, which, mingling with the saliva, is carried with it and the food into the stomach, and contaminates the whole economy.

Rupture of the membrana tympani is a frequent effect of the commotion produced by the discharge of artillery. Sometimes this occasions deafness —sometimes only dulness of hearing.

Balls sometimes lodge in the maxillary sinus, where they are felt by the patient moving about. In 1814, a lieutenant-colonel had had the masseter muscle and parotid gland destroyed by a ball, which had entered the sinus. Excessive swelling supervened; and when this had become diminished, the right nostril remained obstructed, and the sinus the seat of pain. These symptoms were supposed to depend on the presence of fragments of bone. Some were actually removed by operation; but a probe then detected the presence of a ball, which was removed by making an opening into the sinus, between the cheek and the alveolar process.

In wounds of the anterior part of the throat, whether suicidal or otherwise, M. Dupuytren has for some time adopted the use of sutures. He is careful, however, to leave a space, through which either blood or pus may issue.

M. Dupuytren's remarks on gun-shot injuries of the shoulder, or rather of the scapulo-humeral region, are perspicuous, instructive, and characteristic of his style. It is only necessary to particularize one point. When the head of the humerus is shattered, amputation at the shoulder-joint is usually required. Yet, in many instances, the operation of cutting down upon the bone, and removing merely the broken portions, is sufficient to preserve the limb and the life. This is, in fact, the application of the operation of excision of the heads of bones to the case of injury by gun-shot. The operation, in the instance of the shoulder-joint, may be performed by means of a simple incision through the deltoid, or by making a flap of that muscle.

Two facts, in reference to gun-shot wounds of the arm, are briefly mentioned by M. Dupuytren. Of course he indulges in other observations, to which we do not deem it necessary to allude. The facts in question are these.

1. A man received a gun-shot wound on the anterior part of the arm, at its upper and internal part. No nerve would seem to have been injured—at least no paralysis occurred; but a frightful hæmorrhage succeeded, and was only arrested by a ligature.* From that time forwards, no pulsation could be felt in the brachial, the radial, or the ulnar arteries. Those vessels were distinguished filled with blood, but without any pulsatory impulse. It is probable that, in this case, the brachial artery was wounded; but, although the circulation was maintained by the anastomosing branches, it was not sufficiently vigorous to transmit pulsation.

2. The question of re-union of divided nerves occupies the attention of the lecturer. He abstains from the expression of opinion, and leans on the statement of a fact. In two instances, the radial nerve was divided by a cutting instrument. Sensibility and motion returned in the parts supplied by it at the expiration of two years. The reader may turn, with some advantage, to the experiments and the remarks of Mr. Swan.†

In describing wounds of the chest and abdomen, M. Dupuytren alludes to the occurrence of great destruction of the viscera contained within these cavities, although their parietes display no lesion. He relates a case of such contusion of the viscera of the abdomen which occurred in a soldier, wounded in 1814 beneath the walls of Paris. A cannon-ball struck the left flank obliquely, but produced no external wound. The patient, who was carried to the ambulance at Veltette, was rallied by his comrades on his quitting the field of battle. The integuments of the abdomen became discoloured and dark—insensibility and immobility of the left lower extremity succeeded—vomiting, hæmaturia, and dyspnœa followed—and stupor closed the scene. On examination after death, the subcutaneous cellular tissue, the sacro-lumbalis muscle, the parietes of the abdomen, and the left kidney, were reduced to the state of bouilli—the lumbar nerves were torn—the transverse processes of the lumbar vertebræ, and the lower ribs, were shattered—and the cavity of the abdomen and left side of the chest were filled with black blood. The skin alone had resisted the disorganizing action of the shot.

We have been favoured by a Gentleman, whose labours and whose judgment are constantly the means of enriching the pages of this Journal, with

---

* Where, when, or how applied, we are not informed by the lecturer or reporter.

† For an account of those, see the last Number of this Journal.

a notice of a work lately published at Paris, and entitled a Treatise upon Gun-shot Wounds, founded on the clinical lectures before us. The present article upon the subject precludes the entire insertion of our correspondent's; but, as some of the questions are treated more fully in the Treatise than in the lecture, we shall take the opportunity of dovetailing some of the matter of our correspondent with our own. This will account for what might otherwise appear incongruous to those who possess the Léçons Orales. We shall select, in the present instance, the Baron's treatment of wounds of the intestines, for insertion.

"The principle upon which all rational attempts to re-unite wounds of such viscera as the intestines is founded, is the bringing into contact, and retaining in apposition, surfaces which, when inflamed, have a natural tendency to become agglutinated. Now it is well known that mucous membranes have not this tendency, but that serous membranes have it most characteristically. It is on this principle that the proposal of M. Dupuytren, to cure false anus by the section of the "parois adossées de l'intestin" (the lips of the wound being inverted into the cavity of the gut, so that the opposite serous surfaces are brought in contact, and placed, as it were, back to back), is founded; and that, more lately, M. Jobert de Lamballe has been led to "adosser, et maintenir adossée," by means of a suture, the serous membrane of the intestines, for the cure of wounds of these viscera. In the cases for which this description of suture is designed, the extent of the injury inflicted may be various: the gut may be merely wounded at one part, and this wound may be either parallel or oblique to its axis, or it (the gut) may be divided in a perpendicular direction, either partially or entirely round all its circumference. Hence there are different operations to be adopted, according to the nature and severity of the case.

1. When the wound of the intestine is a simple cut, whether the direction of this be parallel or oblique to its axis, we almost invariably find that it (the intestine) becomes more or less contracted upon itself, and that the lips of the wound are more or less turned inwards. If such be the state of parts, we should avoid disturbing it, as the very object of the operation is to bring and retain in contact the opposite and inverted serous surfaces of the wound. We should, therefore, be satisfied with merely washing the wound, and then, with the handle of the scalpel or with the point of the finger, smooth and make even its two lips:—The number of needles to be employed must correspond with the number of the sutures which we propose to make—they are to be passed through the whole thickness of the walls of each inverted intestinal fold (which is necessarily double) and the ligatures are to be tied separately; all the loose ends of the threads may be cut off, and the bowel be then gently re-placed in the abdomen, as near as possible to the outer wound. M. Lembert has observed, in his practice, that the ligatures usually escape into the cavity of the gut at the end of seven or eight days, after having cut across, by ulcerative absorption, the parts which they had embraced, and that a plastic exudation, which becomes very quickly organized, and remains for a considerable time afterwards, forms a bond of union (in the same manner as the provisional callus does in cases of fractured bone) between the wounded and the adjacent textures. The credit of first recommending and practising the above method of operation belongs to M. Lembert.

2. The proposal of M. Jobert is indeed in many respects very similar to that of M. Lembert; the essential difference consisting in his mode of tying the ligatures; for instead of tying each separately, he recommends that all the loose ends should be gathered into two little bundles and these then tied together: the gut being afterwards reduced into the abdomen, the two ends are to be secured to the lips of the outer wound by means of a strip of diachylon, and only when the adhesive union is sufficiently advanced are they to be gently drawn at. M. Jobert's own words, in his Traité Theorique, et Pratique des Maladies Chirurgicales du Canal Intestinal," 1829, are as follow:—

"Pour executer ce procédé, on lave les bords de la plaie avec de l'eau tiéde, on les renverse en dedans avec l'aiguille, et on passe des fils transversalement dans les bords, en ayant soin, qu'ils soient assez rapprochés, pour que les parties qui se trouvent dans l'intervalle ne fassent point hernie, et que les sereuses restent en contact immediat. Les fils sont ensuite ramenés au dehors, et maintenus suivant le procédé de Ledran."*

This operation is however not so praiseworthy as M. Lembert's; the opposite surfaces of the wounded gut being apt to become puckered and irregular, by the ligatures being tied all together.

3. M. Dupuytren proposes a modification of M. Lembert's method; the lips of the wound being brought in contact by their peritoneal surfaces, as already described, he passes through each inverted fold, a single needle, holding one thread, alternately from one side to the other, so that the thread is at every turn carried over the lips of the wound; or, without previously inverting the lips of the wound, "on traverse l'intestin à deux lignes d'une de ces lèvres, et d'un seul trait d'aiguille de dehors en dedans, et de dedans en dehors, on le traverse de même sur la lèvres opposée, et on ramene ensuite, chaque fois, le fil d'un coté a l'autre, de maniére à former au dessus des lèvres de la plaie, dont les bords se trouvent alors renversés en dedans, une espece de spirale, comme dans la 'suture du Pelletier.'" In this suture, the lips of the wound are retained in contact, not only at those points through which the thread passes, but also through the intervening spaces, in consequence of the spiral direction which it follows:—When the suture is completed, the ends of the thread are to be secured on the outside of the wound, as in M. Jobert's operation. The advantage of Dupuytren's method is, that only one needle and one thread are required for its performance.

When the intestinal tube has been fairly divided, M. Lembert recommends that we should employ the same method of operating as in simple or partial wounds; the edges of each divided portion are to be first gently inverted, and their serous surfaces brought and retained in apposition with each other by means of the suture "a points séparés;" much skill is required to adjust the two portions of intestine, but this will be facilitated by previously tying together the separated portions of the mesentery. Dupuytren prefers the "suture du Pelletier" to the interrupted suture employed by M. Lembert;

* The suture of Le Dran consists in gathering all the ligatures on one side of a wound into a bundle, and then those of the other, and tying, or twisting the two bundles together.

and he thinks that this preference is amply justified by the circumstance of M. L. having of late sometimes adopted it. An important rule to be attended to in all cases, is that the first stitches of the suture should be made through the part of the gut nearest to the insertion of its mesentery; when the suture is completed, the gut is to be replaced, and kept close to the outer wound by means of the two ends of the ligature, for five or six days; after which period they may be gently drawn at, to encourage their separation. Whatever kind of suture be employed, we must keep in mind that the success of the operation depends upon the nice apposition of the serous surfaces of the divided intestine. It is not necessary that we should be able to distinguish the upper, or gastric, from the lower, or anal, extremity of the intestine; and this circumstance alone is sufficient to induce us to prefer, in almost all cases, the "suture par adossement" to the "suture par invagination;" for in this latter operation, as the name implies, one step is to insert, or invaginate the upper into the lower portion of the gut, before we proceed to stitch them together: we have no doubt that it will be henceforth banished from surgery; the proceedings necessary for its accomplishment are too numerous and complicated, and tend to excite that very mischief, *viz.* inflammation of the bowels, which is so generally the cause of death in such cases.

It is singular to observe how the intestines sometimes escape injury in penetrating wounds of the abdomen; it can be explained only by supposing that the point of the instrument glides off from their slippery rounded surfaces.

The following is a good illustrative example of this accident:—

M. N. in a fit of severe grief, resolved to put an end to his existence, and for this purpose rushed with all his force against the point of a sword, which he had previously made fast in the wall of his apartment. So completely was the abdomen transfixed, that the point of the sword stuck out for eight or ten inches on the right side of the vertebral column. When M. Dupuytren saw him, he seemed to suffer but little pain; but this might arise either from the insensibility so common in all cases of suicidal mania, or from the circumstance of the instrument not having wounded any of the principal nerves. On examination, there were fortunately no symptoms of any extravasation, nor indeed of a wound of any of the abdominal viscera. It required considerable force to withdraw the sword, and Dupuytren was afraid that some hæmorrhage or extravasation might follow the operation; happily no such accident occurred, and the patient, after repeated bleedings and the employment of a very rigid antiphlogistic regimen, was speedily quite cured."

M. Dupuytren's remarks upon incisions are good, yet we know not that any would be novel or striking to the English surgeon. The papers of Mr. Hutchison, Mr. Lawrence, and others contain, perhaps, more than the Lectures of the Baron, on this important remedy.

M. Dupuytren offers a remark on the merits of English lint and of French *charpie*, to which we may direct a moment's attention. He observes that, in gun-shot wounds, where union by the first intention is neither desirable, nor to be obtained, the charpie is preferable as a dressing to our lint; for, the former becomes saturated with the matter which is formed, while the latter tends to confine it. The remark is very just, and the greater use of charpie in this country, would, we think, be an improvement in our practice. Wherever suppuration is copious, and poultices not applicable, the charpie of the French forms an admirable dressing. The best substitute with which we are acquainted is lint, pulled into shreds.

M. Dupuytren speaks slightingly of the chlorides, or chlorates, as a local application in cases of gangrene. We cordially agree with the experienced lecturer; and we really believe that, much as they have been vaunted, they are possessed of little power in any kind of sore. The diluted chlorate of soda or lime is a gently stimulating, and a very cleanly lotion. We fancy this is a tolerably fair expression of its merits.

The feeble therapeutics of France are displayed in M. Dupuytren's incidental advice with respect to the treatment of jaundice. He merely recommends fomentations on the region of the liver.

We see little in the remainder of this series of clinical remarks to attract our attention, or occupy our space. A large portion is exclusively devoted to the consideration of the treatment of tetanus. That nothing new is said, and nothing satisfactory suggested, may probably be expected, and is really the case. M. Dupuytren condemns the practice of amputation, after the tetanic symptoms have appeared. He relates some cases which display its inefficiency, a fact of which the majority of surgeons are sufficiently aware.

We may now proceed to the third volume of these lectures. It contains eighteen articles, devoted to the following subjects:—Cysts developed in the substance of bones—serous cysts, containing small white bodies—the nail growing into the flesh—dislocations of the humerus—vital and mechanical dilatation of the urethra—club-foot—laceration of the perinæum during labour—congenital dislocation of the femur—fistula lachrymalis—ulcer of the rectum—ranula—abscess in the right iliac fossa—the modes of employing the actual cautery and moxa—hydatid tumors in the muscles and the viscera—fracture of the lower extremity of the humerus—exostosis of the superior surface of the last phalanx of the great toe—fibro-cellular tumors of the uterus—tracheotomy.

Some of the preceding subjects are of great, and some are only of minor importance; some have been noticed on former occasions, and some are probably not deserving of particular attention. We proceed to discuss them in their proper order, and shall endeavour to select important particulars, unencumbered with the superfluous or familiar details of a lecture.

### ON THE CYSTS DEVELOPED IN THE SUBSTANCE OF THE BONES.*

This is really an interesting, and rather an instructive lecture. The object of the lecturer is to point out the circumstances which constitute the difference between the simple and non-malignant cysts developed in bones, and

---

* In our analytical notice of these Lectures, we shall adopt a free and liberal interpretation of the text. The translator should allow not merely for difference of idiomatic language, but for national peculiarities of sentiment. The *same* idea may be differently conceived, as well as differently expressed, by individuals of different nations.

the genuine malignant diseases of their tissue. The reporters congratulate themselves and their readers on the perfect success of their patron. Of this we are not so perfectly assured; but our surgical brethren may decide for themselves.

The bones are the seat of cysts, whose parietes are extremely thin, and composed of bony matter, resembling in its tenuity hammered metal. The contents of the cysts are various—most frequently they consist of a fibro-cellular substance—sometimes of a serous fluid, unmixed or united with the latter—sometimes of a mucous fluid—or of adipocire—or hydatids—or pus and serum—or a jelly-like substance—or teeth—and so on. M. Dupuytren relates instances of some of these varieties; but we need not do more than enumerate them.

These cysts are seated in the substance of the bones. They are seen in the extremities of the long bones, in the bodies of the vertebræ, but most of all in the bones of the face. They are frequently displayed in the horizontal portion of the lower maxilla, in its ascending ramus, in the alveolar process of the superior maxillary bone, in the antrum of Highmore, and in the nasal fossæ. Their form is generally ovoid, occasionally oblong, and it may be flat. Their size is varied, no larger than that of a musket-ball on one hand, and equal to the dimensions of a fist upon the other. The parietes of these cysts are formed at the expense of the bone, in the interior of which they are developed.

The circumstances which give rise to these osseous cysts are in general obscure. External violence, as a blow, has preceded their formation—so has the incomplete extraction of a carious tooth. M. Dupuytren makes some remarks on the influence of the teeth on the production of the cysts. He observes that the latter are occasioned by alterations in the roots of the teeth, and are then most commonly developed in the alveoli of the canine of the upper jaw, where sometimes they attain a considerable magnitude. If, under these circumstances, the affected, or rather the affecting, tooth be examined, its extremity is found altered, surrounded with an osseous deposit, and bathed in the liquid contents of a bony cyst, attached on the one hand to the osseous deposition on the tooth, and, on the other, to the bottom of the alveolar cell. This cyst in general follows the tooth, when the latter is extracted; if left behind, it occasions tedious suppuration. Its contents are a liquid, sometimes very thick, and sometimes serous, and its internal lining membrane is smooth as serous membranes are.

We pass from this imperfect enumeration of the causes, to the symptoms and the diagnosis.

The earliest symptoms are uneasiness or pain; the latter is sometimes acute, and sometimes dull, but rarely of a lancinating character. After an interval, swelling commences, and a tumor of variable size is formed. This is dependent, as has been remarked, on the separation of the laminæ of bone by the cyst. The bony parietes of the tumor are so thin, that they yield beneath the pressure of the finger, and communicate, in yielding, a crackling sensation, resembling that produced by dry parchment. This symptom or sign is regarded by the Baron as pathognomonic. After frequent examination and repeated pressure, it will sometimes disappear, a circumstance which is probably occasioned by the elasticity of the lamina of bone being overcome, and by the lamina itself being temporarily, or even permanently depressed. In this, as in other tumors, exploration by a puncture is always advantageous, in removing doubt or increasing certainty.

The disease with which the osseous cyst is most liable to be confounded is osteo-sarcoma. M. Dupuytren consequently bestows some pains in laying down their distinctive characters.

Osteo-sarcoma, he observes, is distinguished from its commencement by lancinating pains—enlargement of the veins—simultaneous implication of the neighbouring parts, whether soft or hard—their disposition to form a fungus, and to constitute a tumor irregular upon its surface. On the other hand, the osseous cysts do not contaminate the surrounding parts ; their surface is smooth, and their increase is slow—that of osteo-sarcoma being rapid. The latter kind of tumor is traversed in its interior by spiculæ of bone ; this is never observed in the osseous cyst. When we add to these diagnostic characters the crackling of the osseous cyst under pressure, and the information which a puncture can afford, we have run through the principal features of both maladies.

M. Dupuytren adds the following summary.—1. Osteo-sarcoma and the osseous cysts differ, in essential respects, from each other. 2. Osteo-sarcoma is a cancerous affection of the bone ; but the cyst is a development of it or in it, owing, in most instances, to the presence of a fibrous body, resembling the fibrous tumor of the uterus. 3. The osseous cyst is seldom or never reproduced, when once completely extirpated ; osteo-sarcoma, like other malignant tumors, most commonly returns after any kind of operation. The osseous cyst is usually, as has been stated, slow in its progress—sometimes, however it is rapid, and sometimes it may even be stationary. After an uncertain period, the osseous cyst is convertible into a malignant structure, especially into such as exhibits a fibro-cellular character.

The facility with which the osseous cyst is replenished or regenerated, when merely opened, or imperfectly removed, is displayed by one remark, and two conclusive cases. Before we pass to the prognosis and the treatment, we shall venture to offer one or two observations. It appears to us, that M. Dupuytren deserves our thanks for drawing attention, in so marked a manner, to the frequent existence of cysts, or encysted tumors, in bones, which, however serious, are not so malignant as genuine cancer. Most hospital surgeons were, we fancy, aware that diseases of this nature occasionally arise, and that operations for them are more successful than in cases of scirrhus or fungus hæmatodes. The maxillary bones have been partially, or even totally, removed by English and by foreign surgeons, for cysts of the nature described by M. Dupuytren ; and probably few museums are deficient in examples of it.* Yet we do not feel convinced of the strict propriety of the diagnostic marks laid down by the Baron, nor are we quite assured that it is always so easy as the reporters hint, to draw the distinction between the non-malignant osseous cyst, and the really malignant fungoid tumor. We recollect the case of a young woman, who was under Sir Benjamin Brodie's

* There is a splendid specimen of this affection of the ascending ramus of the lower jaw in the surgical museum of St. George's Hospital. The disease was removed in a masterly manner by Mr. now Sir B. C. Brodie. The patient died of erysipelas. The cyst is chiefly cartilaginous or fibrous, with here and there scales or patches of ossific matter.

care in St. George's Hospital. There was a tumor of the upper jaw, which protruded in the situation of the antrum, encroached upon the nose, and projected in the mouth, and displayed, in a very characteristic degree, the crackling alluded to by M. Dupuytren. A puncture was made by Mr. Brodie, but blood only was discharged, and other circumstances made it too probable, that the malady was fungus hæmatodes.

One of the cases related by M. Dupuytren himself is calculated, we fear, to confirm our suspicions.

*Case.* A young girl, about seven years of age, was taken to the Hôtel Dieu, on account of a tumor in the superior maxillary bone. It was equal in size to the fist—the right nostril was obstructed, the eye protruded forwards, and the palatine arch pushed upwards and aside. The disease had followed a blow upon the cheek, some time after the reception of which pains had been experienced, and swelling had succeeded. The child had decidedly lost flesh. M. Dupuytren was inclined, in the first instance, to believe that the disease was osteo-sarcoma. But the discovery of "the pathognomonic symptoms" of an osseous cyst relieved his alarm and dispelled his suspicions. On compressing the anterior and superior portion of the tumor, it first yielded, and then returned to its original condition, with the crackling similar to that of parchment. The same thing was observed in the bony palate. M. Dupuytren was re-assured. He made an incision into the tumor, through the mucous membrane of the lip—black blood only escaped. He introduced the finger through the wound—it passed into a soft substance, easily torn, which had expanded the bone, but was not confounded with it; in fact, the finger distinguished the existence of osseous parietes, firm in some parts, thin in others. On the next day, the patient was carried to the operating theatre, and an incision was made into the most dependent portion of the tumor. About two ounces of blood escaped, and M. Dupuytren detached with the finger a portion of the substance that filled the cyst. Injections were subsequently used, and, in ten days after the performance of the operation, the tumor was greatly diminished in size. M. Dupuytren hoped that, *if* the tumor continued progressively to decrease, a cure might be effected.

We fear that the English surgeon would regard this case as one of malignant tumor, the escape of blood only when a puncture was made, and the soft contents of the expanded bone, presenting two of the important features of fungus hæmatodes. We feel disposed, as we have stated, to doubt the strict justice of the distinction, which M. Dupuytren has laid down, between the non-malignant cyst and the malignant tumor. We fear the transition is too gradual to permit the abrupt and decided line. Yet still the remarks of the Baron are valuable, and, if they cannot be received implicitly, they are at least a fair approximation to the truth.

We now proceed to the treatment of these osseous cysts. Their destruction is required.

In the greater number of cases, it is necessary, in the first instance, to puncture the tumor, in order to ascertain the actual nature of the contents of the cyst. This having been done, an incision must be made into the tumor, and, if the cyst is seated in the face, the inside of the mouth should be selected for the wound. If the contents are solid, they should be extirpated, and the use of the actual cautery is frequently demanded. The contents of the cyst having been removed, the latter should be dressed in freely with lint, and emollient or irritant injections should be employed, as circumstances may require. Inflammation of the parietes of the cyst is thus produced, and the membrane which lines it is destroyed; after this, the parietes contract upon themselves, and a cure is effected after a longer or shorter space of time. In some cases, it is necessary to make a counter opening, and to carry a seton between the two wounds.

Such is M. Dupuytren's treatment of these osseous cysts. We have doubted if they may not be more frequently malignant than M. Dupuytren supposes, and we doubt, in continuation, if the treatment he advises will be commonly efficient. We are sure that, in an instance of osseous cyst of the lower jaw, which we have witnessed, the attempt to cure the malady by opening the tumor, and exciting inflammation of its interior, would have almost been preposterous. But we bow to the Baron, and proceed to the next article.

### ON SEROUS CYSTS, CONTAINING LITTLE WHITE BODIES IN THEIR INTERIOR.

The analysis of Mr. Brodie's work, which forms the article, in our present Number, immediately succeeding this notice of M. Dupuytren's lectures, displays the sentiments of Mr. Brodie on inflammation, and other diseases of the mucous bursæ. In that article will be found Mr. Brodie's description of numerous small bodies, resembling melon-seeds, which the bursæ frequently contain.*

When the inflammation of a bursa mucosa, says Mr. Brodie, is of long standing, it is not unusual to find floating in the fluid of the bursa a number of loose bodies, of a flattened oval form, of a light brown colour, with smooth surfaces, resembling small melon-seeds in appearance. There seems to be no doubt that these loose bodies have their origin in the coagulated lymph, which was effused in the early stage of the disease; and Mr. Brodie has traced the steps of their gradual formation, from irregular masses of no determined shape, to smaller portions, and, ultimately, to the flat, oval bodies described. Motion and pressure are the agents of change.

Such is the passage which our readers will find a little further on, in our review of Mr. Brodie's admirable volume. These peculiar substances contained within the bursæ, or, as M. Dupuytren terms them, in serous cysts, form the subject of the lecture now before us. We shall not pursue the lecturer through his lengthened cases and his trivial details, but select only such as are necessary to supply a consistent account of the complaint.

He observes that, before M. Cruveilhier published his Essay on Pathological Anatomy, very little was known respecting this affection. On this, as on many other occasions, M. Dupuytren has displayed his profound ignorance of the state of surgical science in this country. Mr. Brodie and Mr. Brodie's work had rendered the profession familiar with the complaint long before M. Cruveilhier's Essay saw the light. Nay more—we shall

---

* *Vide* page 350 of this Number.—EDS.

soon shew that M. Dupuytren's ideas upon the subject are much less rational than those of Mr. Brodie; for he (M. Dupuytren) actually believes that the small white bodies are genuine hydatids, whilst he does not appear to be properly aware that the " serous cyst" is no other than a bursa.

M. Dupuytren observes, that the disease is usually remarked on the palmar surface of the wrist, beneath the anterior carpal ligament; sometimes however, it is noticed on the ankle, beneath the anterior annular ligament; and, in some rare cases, it has been observed on the olecranon—on the acromion—situated over the tuberosity of the ischium—and behind the great trochanter.

Falls, blows, pressure, distention, and repeated friction, are the usual causes of the malady.

M. Dupuytren's description of the foreign bodies is minute. He says that they are whitish, transparent, folded in their long diameter, forming a kind of pouch, of which one extremity is terminated by a broad and rounded cul-de-sac, whilst the other is elongated like the neck of a bottle, and finished off in the form of a sucker or mouth! They are evidently composed of laminæ, super-imposed upon each other. Their form is sometimes nearly cylindrical—sometimes conoid—and sometimes it is lenticular. Some are small, others large, and they seem to pass through several phases before they arrive at their mature development. Their consistence is almost cartilaginous; but M. Dupuytren thinks he has discovered a cavity in their interior. These whitish bodies are inclosed in a thin, smooth, yellowish, and serous cyst, containing transparent serosity.

Some of these bodies were sent to M. Bosc, a member of the Institute, in order to be submitted to a careful examination. He arrived at the conclusion, that they were not hydatids, nor any other description of animal, but merely debris of fatty cellular tissue, swimming in serous fluid. M. Dumeril arrived at the same conclusion.

M. Dupuytren remarks, that it is easy to convict these naturalists of a blunder. For, says he, when they are compressed (the little bodies, not the naturalists) between two portions of blotting paper, no appearance of grease can be observed. Their constancy of form and their lamellated structure induce him to believe, that they enjoy a distinct and individual existence, and he thinks, what would be very important if true, that he has seen several of these bodies move.

We need scarcely say, that we believe M. Dupuytren to be totally wrong, and Mr. Brodie and the naturalists to be right. We know that Mr. Brodie has carefully and often examined these bodies—that many others have examined them too—and that their firm and unanimous opinion has been against their hydatid nature. We have frequently regarded them with a lens ourselves, but never did we recognize mouth, or cavity, or motion. M. Dupuytren's microscope must, we fancy, be a good one, to allow him to perceive these conclusive properties.

There are two circumstances connected with the symptoms, on which M. Dupuytren lays especial stress.

The first has reference to the form of the cyst. It is always bisected in the middle, so as to constitute a double tumor, the portions of which are nearly equal. The second symptom is this—pressure upon either causes the fluid to pass into the other portion, in doing which, a kind of crepitus is evident. This is the pathognomonic sign of the presence of these bodies in the cyst.

The Baron's statement is partially true, and partially erroneous. The tumor is not always bisected. M. Dupuytren does not appear to be aware that any bursa may contain these substances. We have seen them in the bursa over the patella. The division of the tumor into two portions is the consequence of the disposition of the neighbouring parts. The bursa on the palmar aspect of the wrist extends, as is well known, above and below the transverse ligament. When distended by effusion, the unyielding ligament binding it down gives rise to the projection above and below. The same thing occurs on the front of the ankle. But over the patella no tendon nor ligament can exert compression, and there the tumor is globular or oval. Yet the crepitus is as distinct as in the wrist or on the ankle. We lately saw an instance of enlargement of the bursal sheath investing the internodial muscles on the posterior aspect of the radius. The tumor was irregularly oval, but presented no contraction in any part. The peculiar crackling attendant on the presence of the melon-seed-like bodies was remarkably distinct. We punctured the tumor and they freely issued from the opening. From the preceding observations it will, we think, be apparent that the Baron has committed a serious mistake, in considering division of the tumor into two portions as a feature invariably present. He has also, it would seem, been guilty of an omission, if not of an error, in not mentioning the fact that the "serous cysts" are usually no other than enlarged bursæ, or bursal sheaths investing the tendons.

The remaining remarks to which we shall direct attention are important, because connected with the treatment.

M. Dupuytren insists on the necessity of a large opening. When the tumor is bilobed, the incision should be made into either half. The necessity of a free incision is apparent when we recollect the anatomical relations of the cyst. On the ankle, and especially on the palmar surface of the wrist, the cyst is seated beneath the fascia, in the midst of tendons, vessels, and nerves, and surrounded by a fibrous cellular tissue. If a small opening is made, inflammation and suppuration occurring in the cyst give rise to swelling: to strangulation by the fascia and the tendons: to diffusion of matter along the sheaths of the tendons and the vessels in the hand, the fore-arm, and the arm: and to all the consequences which such mischief may occasion, among which death itself must be enumerated. When a large incision is practised in either half of the cyst, such disastrous results are less likely to ensue, for the matter which may form obtains a ready exit.

These free incisions having been performed, and the fluid and small bodies contained within the cyst having been set free, a portion of lint is to be introduced between the lips of both the wounds.

M. Dupuytren was once in the habit of using a seton, which passed from one to the other opening. This, however, he found so dangerous, that experience compelled him to abandon it. The inflammation that ensued was violent and extensive, and yet the hazard was unattended by any corresponding quantum of advantage over the simple introduction of lint. We may notice a case related by the Baron, in order to display the perils of the seton. We have lately witnessed a fatal result from the use of the same means, in an instance of enlarged bursa over the inferior extremity of the scapula.

*Case.* A carpenter, æt. 35, first observed a tumor on the anterior aspect of the right wrist in the early part of 1813. It was a serous cyst, compressed about its centre by the transverse carpal ligament. It produced such inconvenience, that in June, 1814, he placed himself under the care of M. Dupuytren. The Baron made an incision in each portion of the tumor, discharged a number of the melon-seed-like bodies, divided the fascia so as to diminish the risk of strangulation, and passed a seton between the two openings. Severe pain ensued in the night succeeding the operation—swelling followed, and increased till the fourth day—and dirty-coloured, flaky suppuration was established. On the fifth day the seton was removed. The inflammation spread up the arm to the axilla, and the general symptoms became severe. On the eighth day, the sloughy fascia was divided—an abscess between the first and second metacarpal bones was opened—and compression was applied to facilitate the discharge of matter which extended to the hand and up the forearm. On the tenth and eleventh days there were rigors—irremediable prostration followed—and the fifteenth day was the patient's last.

M. Dupuytren concludes the article with an injunction not to urge the performance of an operation, no matter of what kind, unless the disease be extremely inconvenient. Under any circumstances, the patient should be told of the risk that waits on surgical interference. It would be equally foolish and improper on the surgeon's part, to promise at the same time immunity from danger, and certainty of cure. It must be owned that operations on the bursal sheaths are always attended with considerable hazard. The superficial bursæ may be meddled with more safely.

### On the Nail growing into the Flesh.

The designation of this complaint is not perhaps unexceptionable or correct. It is, however, understood, and that will answer our purpose for the present.

M. Dupuytren remarks that, looking on the malady with a curious eye, he soon discovered the existence of two important varieties, requiring each a peculiar mode of treatment.

The first variety consists of ulceration of the skin at one of the lateral borders of the nail, or even in some instances at both; when on one, it is usually on the outer side. A little reflection will readily explain how a light or ill-made shoe may press the edge of the nail into the skin, and produce inflammation, with its consequences. Such is, in fact, the general mode in which the affection is produced.

The complaint almost always commences at the angle where the lateral and anterior border of the nail unite. This appears to M. Dupuytren to be owing to individuals neglecting or avoiding to meddle with that angle when cutting their nails. Allowed to grow, almost undisturbed, it constitutes a point which runs into the flesh, and gives rise to the commencement of an ulcerative process, that subsequently extends the whole length of the lateral border of the nail.

Of the symptoms of this very painful affection we think we need not speak. They are usually too obvious to escape the observation, or deceive the judgment of the patient or the surgeon. Yet M. Dupuytren relates a case, in which they were mistaken and treated for gout, during the lengthened period of eight years.

M. Dupuytren reviews the principal methods of treatment adopted, and concludes by enumerating the superior advantages possessed by his own. We shall disregard his criticism, and attend to his advice.

After diminishing by fomentations and by other means the general inflammatory condition of the part, the Baron passes the point of one blade of a pair of scissors under the centre of the free edge of the nail. By a rapid thrust the blade is carried back as far as the root of the nail extends, and the scissors are made to divide the nail into two lateral halves. That corresponding to the side affected is then seized with a pair of dissecting forceps, and torn or twisted off by a kind of rotatory motion, directed from the centre of the nail towards its side. If the other side of the toe is affected, the other side of the nail is removed. If fungous granulations exist, they are liberally touched with caustic. Appropriate dressings are afterwards applied, and cicatrization rapidly ensues. In general, the nail is not reproduced in old persons, but sometimes it is found to be regenerated in young ones.

The kindly eye of parental attachment is obvious in the following declaration. It *might* be supposed, observes the Lecturer, that the treatment just described would be very painful; but patients, notwithstanding, seldom cry out, when compelled to undergo it. If not a word was ever uttered, we think it would be difficult to convince one human being, that slitting up his toe-nail with a pair of scissors, and forcibly twisting off either half, is not most intolerable anguish. Whatever M. Dupuytren may believe, his process is abominably violent and painful.

We have treated many cases of this complaint, and we never employed, nor do we think we would advise, the treatment resorted to by M. Dupuytren. We have not in any instance failed to remove the complaint effectually, by means of the following more gentle management. The affected side of the nail must be scraped, in order to render it as thin as possible. The fungous granulations should then be touched with caustic, for the double purpose of affording room and diminishing their excessive sensibility. These preliminaries having been adopted, the surgeon should gently and gradually raise the edge of the nail, with the flat end of a small eye-probe. The loose and elevated portion is to be removed by means of a pair of dissecting scissors. The surgeon must proceed with this process of raising the nail and removing it, until he gets beyond the existing fungus, and arrives at the connexion of the nail with the sound cutis. Here he is to stop, for where the fungus is, the nail is necessarily separated from the cutis: and where the latter is connected with the nail, there can be no fungus, and consequently no disease. The remaining fungus should now be freely destroyed with lunar caustic, and dry lint should be applied. At the subsequent dressings the nail should be gently and gradually loosened from the cutis, so as to admit dry lint beneath its edge, which is to be cut and kept cut, in order to prevent its approaching the wound, or even the cicatrix. Such is the method which, in our own case, as well as in that of many others (for in hospital practice the complaint is common), we have found attended with little pain and with complete success. No doubt M. Dupuytren's is equally successful, but then it is cruelly severe.

The second variety of this affection next engages the attention of the lecturer. His observations on this subject are extremely good, and cannot be materially reduced.

We have seen that, in the first variety, the nail presses in upon, irritates, and inflames the soft parts at its side. In that variety, the initiative is, therefore, seated in the nail. But in the one to which we now proceed, it is the cutis which is primarily diseased, and the nail is affected in a secondary manner.

This affection commences at the base of the nail, in the skin which forms its matrix, and from which it grows. A few words on the anatomy and growth of the nail will, probably, not be misplaced.

The base or adherent extremity of the nail is implanted in the skin in a particular manner. The cutis have reached a little way over the nail upon its dorsal surface, is reflected to the posterior border of the nail, under which it proceeds to form the plane on which the nail is laid. The cuticle, however, instead of passing beneath the nail, is reflected back for a shorter distance than the cutis, and becomes continuous with the external lamina of the nail itself. Thus, the base of the nail is received in a kind of groove of the cutis, which is termed its matrix, and out of which it grows. The variety of disease which we are now considering, is essentially an affection of this portion of cutis. From some cause or other it inflames and ulcerates, the ulceration displaying a semilunar form, with hard and elevated edges, and a deep red, or livid and violet colour. The nail is shortened, and reduced, perhaps, to half its extent, or even totally removed. In its place, some portions of a horny sort of substance are seen to spring up here and there: and frequently a portion of the nail is found concealed beneath the fungous granulations. The latter are, as we observed, confined to the base of the nail and its vicinity.

The colour of the nail, when it remains, is altered to a grey or to a black tint. Sometimes it is loose. The wound is generally bathed in a sanious or bloody suppuration, which, conjointly with the natural secretions of the parts, secretions in these patients always offensive, gives rise to an odour so insupportable, that other individuals are incapable of living with them. The attempt to walk, or even to stand, is productive of great pain, and occasions bleeding of the fungus. Any kind of shoe is perfectly unbearable.

The preceding symptoms are displayed in most cases, with little variation. In a few, however, the disease appears more particularly to affect the portion of the skin which is immediately subjacent to the nail. In instances of this description, this organ is observed to be raised by small tumors, of a fibrous, cartilaginous, osseous, or, perhaps, of a vascular nature, which give rise to suffering, in proportion as the pressure upon them is considerable.

The treatment required for the first variety of the complaint is quite insufficient for the removal of this. The disease is in the cutis; the removal of the nail will not remove that which the presence of the nail has not occasioned. In short, the affected portion of the cutis must be thoroughly dissected out. The measure is severe, but it is necessary.

M. Dupuytren puts his patient on a bed or chair, and makes with a scalpel a deep semicircular incision, three lines, or thereabouts, beyond the matrix of the nail, parallel to which it is directed. The affected portion of skin is then dissected off, by proceeding from behind forwards, the nail, if it remains, being necessarily removed with it. If any pieces of a horny substance still exist, they also are successively removed, till not a vestige of a morbid structure can be seen. All the white-looking and fibrous portions, remarked at the bottom and towards the angle of the wound, should be carefully removed, for otherwise they may reproduce the nail, and re-establish the disease.

Mild dressings should be afterwards applied, and the usual means to avoid inflammation, afford relief to pain, or expedite the healing process, should be had recourse to. If any appearances of horny matter are at any time presented, the knife must immediately remove them. After cicatrization, the skin, when examined, is found to be smooth, but thick, and sometimes of a horny consistence. In conclusion, M. Dupuytren offers the following brief resumé.

1. In general, when the nail is the seat of alteration, it presses injuriously on the neighbouring skin, and constitutes the disease denominated—"the nail growing into the flesh." The removal of the nail is the only successful method of treatment.

2. The disease which is characterized by inflammation, in the first instance, of the skin which serves as matrix for the nail, is distinct from the preceding in its symptoms, its results, and the treatment which is necessary. The best and the quickest method of cure is to remove the whole diseased skin with the knife.

3. In every case of either disease, we must not lose sight of antiphlogistic or other remedies, which, according to circumstances, are always of service, and may sometimes obviate the necessity of resorting to a painful operation.

### On Dislocations of the Humerus.

The able work of Sir Astley Cooper has made the subject of fractures and dislocations too familiar to the English reader to admit of much further illustration at present. Sir Astley has stated the leading facts so forcibly and clearly, that the vividness of the impression produced by his descriptions is rather diminished than increased by trifling subsidiary details. We shall not, therefore, analyse the lengthened lecture of M. Dupuytren on luxations of the humerus, consisting, as it does, of nearly seventy pages, but shall select such portions as express the result of the Baron's experience on disputed points, or seem to present some novel views, or some hitherto unheeded facts.

M. Dupuytren remarks, that surgeons have denied the existence of incomplete dislocations in ball and socket joints; but that morbid anatomy has displayed their incorrectness. In 1824, the surgeon in chief of one of the hospitals in Paris presented to the Academy a preparation, taken from the body of a man who had died eight months after dislocating the humerus. A new socket had occurred, partly formed by the glenoid cavity of the scapula, and partly by a small portion of the surface of the ribs, whilst the head of the humerus was grooved, to receive the anterior edge of the glenoid cavity. This disposition gave rise to a kind of ginglymus joint. During life, the individual had only the power of slight motion forwards and backwards of the arm. The surgeon who reported this fact to the Academy saw the softened head of the femur, in a case of spontaneous dislocation, lying and fixed on the anterior border of the acetabulum.

The incomplete luxation of the femur, in cases of disease, has, we think, been sufficiently known in this country. But, in instances of that description, the acetabulum and its margins are usually altered. Sir Astley Cooper unless our memory belies us greatly, has described with some minuteness an incomplete luxation of the head of the humerus, which is placed on the margin of the glenoid cavity and the coracoid process. The reported good offices between Sir Astley and M. Dupuytren should have brought this circumstance to the memory of the latter.*

The distinctions between the symptoms of luxation of the humerus into the axilla, and fracture of the cervix of the bone, are sometimes so obscure, as to require much experience and the utmost care to effect their discrimination. The remarks of M. Dupuytren are deserving of attention.

Every one, says the Baron, affected with a dislocation or fracture of the upper extremity of the humerus, has received a fall on the same side of the body.† But the situation and position of the limb, at the moment of the fall, differs in the two cases, and the difference usually decides the nature of the accident that is to follow. If the extended arm has been carried forwards or behind, to break the fall, the consequence is dislocation; but, if the arm has been retained in apposition to the side, and the fall has occurred on the shoulder itself, fracture of the head, or of the upper part of the humerus, ensues.

On this statement we would make one observation. In many, perhaps in the majority of instances, the patient is aware of the manner in which he received the accident, and can remember if he fell on the hand or on the shoul-

---

* The following story of M. Dupuytren and Sir Astley has been told. When the former was in London, and accompanied Sir Astley round the wards of Guy's Hospital, he saluted the worthy Baronet, on parting, in the Continental fashion, with a kiss. Sir Astley blushed, but turning to the laughing students observed, with a wink, that he had had the advantage of the Baron, for, in Paris, he had kissed his daughter. The anecdote is characteristic, and may be true.

† This is not strictly correct. Dislocations of the head of the humerus have occurred in consequence of the application of some sudden extending power to the hand. A boy, for instance has been holding a horse by the bridle, when the animal, suddenly raising its head, has occasioned dislocation of the head of the humerus into the axilla. The rationale must be obvious.—Eds.

der, with the arm close to or away from the body. But it is also far from unfrequent, to find that the accident has been of such a character as to prevent the individual from knowing, or from recollecting, these minute particulars. A man, for example, falls from a height, and dislocates his shoulder. He is bruised in many places, and of course it is impossible for him, or for the surgeon, to determine by the history, or by any trivial difference in the marks of contusion or of violence, whether the humerus has been dislocated or broken. We are convinced, too, that the fact is not always as the Baron states it. We have seen dislocation of the head of the humerus occasioned by direct injury to the shoulder, and we would caution the inexperienced surgeon against attaching implicit confidence to this species of diagnosis. It is certainly an assistance, frequently a great one; but it must not be received for more than it is worth.

To return to the remarks of M. Dupuytren. In the case both of fracture and of dislocation, there is pain in the shoulder, and the patient always thinks that the fall has been upon it. When the pain, however, is occasioned by a dislocation, and the fall has taken place on the palm of the hand, the latter bears evidence of such having been the case, in the shape of dirt, excoriation, or bruises; but if fracture has occurred, and the shoulder itself has been the stricken part, the tearing or soiling of the clothes which cover it, and the marks of injury on the shoulder itself, are equal evidence of the nature of the accident. In dislocation, the pain is produced by the laceration of the fibrous capsule and contiguous parts; in fracture, by the bruises of the shoulder, and the injury inflicted by the lower extremity of the bone.

Ecchymosis is observed in both species of accident. In dislocation, it is comparatively rare, and, being produced by the tearing of the ligament, and by internal injury, it is situated on the inner and anterior part of the arm. In fracture, on the contrary, this symptom is almost invariably present, and is found on the shoulder itself.

In both accidents, the acromion projects, a hollow is felt beneath it, with flattening of the deltoid, and a prominence is felt in the axilla. The projection of the acromion, and the flattening of the deltoid, are greater in dislocation than in fracture, in the latter of which the muscle seems shortened, and, as it were, swollen. The hollow under the acromion is greater, also, in dislocation than in fracture, and the prominence in the axilla is more distinct. In dislocation, that prominence feels rounded—in fracture, it is more irregular.

Mobility of the limb and crepitus do not exist in dislocation, whilst they are easily recognized in fracture. The crepitus is most apparent when the humerus is rotated upon its axis. The experimentum crucis of a fracture or a dislocation is, perhaps, the attempt to effect reduction. After the reduction, the limb is readily retained in its position, in the case of dislocation; but particular care, and an apparatus, are required to maintain it properly when a fracture has occurred.

M. Dupuytren observes that fracture, without displacement, is occasionally mistaken for a violent bruise upon the shoulder. The crepitus and mobility, distinguished by rotation of the humerus at the elbow, afford the only means of diagnosis. The lecturer properly points out a source of fallacy, which frequently misleads inexperienced surgeons. In severe contusions of the shoulder, as of other joints, a kind of crepitus is occasionally noticed, although no fracture has occurred. M. Dupuytren asserts that this is occasioned by inflammation of the articulating surface of the joint, and consequent deficiency of synovial secretion. Now this appears to us to be a bad explanation. For no other symptom of inflammation of the synovial membrane is presented in these cases, and if such inflammation did exist, secretion would probably be increased rather than diminished. A more consistent rationale will be found in the belief, that either blood or lymph is effused in the textures external to the joint, and that motion of these textures gives rise to the phenomenon.

M. Dupuytren relates some cases, but the subject has probably been sufficiently pursued.

The symptoms and the treatment of old, unreduced dislocations occupy the attention of M. Dupuytren, and may not unprofitably attract our own. With respect to the symptoms, little need be said. The diagnosis is sometimes very difficult, and must be principally formed through the medium of a practical acquaintance with the characters of dislocations in their early stage. M. Dupuytren lays particular stress on the following features:—The elongation of the arm—the lengthening of the anterior border of the axilla—deformity of the shoulder, and facility of producing a depression in the deltoid by pressure with the fingers.

The treatment of dislocations of some standing contains the interesting and important problem—what lapse of time is sufficient to render attempts at reduction improper?

Surgeons have differed widely on this point, and the ancient practice is strongly contrasted with the modern. Benjamin Bell, even, was of opinion, that reduction should not be tried, if some days had elapsed after the occurrence of the dislocation. His advice has been contemned by judicious boldness, and the consequence has been, the accumulation of a mass of facts in opposition to it. By those facts, or at least by a portion of them, M. Dupuytren attempts to arrive at some determinate conclusions. Before we proceed to them, it is necessary to clear away an obvious objection that almost unavoidably presents itself to the mind, when reflecting on this subject. We allude to the possibility of injury being inflicted on the vessels and the nerves in the vicinity of a joint, from the violence indispensable to effect the reduction of a dislocation of long standing. That reasonable supposition would seem to be supported by a memoir, published by M. Flaubert, surgeon to the Hôtel Dieu of Rouen. In that memoir are contained some extraordinary instances of mischief and disaster. Some six or seven years ago, we noticed the Memoir in this Journal; but we think it would be well to present, as M. Dupuytren has done, an abstract of the cases related by M. Flaubert. They are calculated, at all events, to read to the incautious or the inexperienced surgeon a lesson of gentleness and prudence.

*Case* 1. The patient was a sturdy sailor, æt. 57. The head of the humerus was dislocated under the pectoral muscle; the dislocation had existed for eleven days. The reduction was effected in the following manner. The patient being seated in a high chair, extension was made from the wrist, whilst the counter-extension was effected by a roller, passed under the arm-pit, the ends crossing over the opposite shoulder, and fixed to a staple in the wall. A ball was kept in the hollow of the axilla, to lift up the head of the bone at the instant of reduction. The extension was made by eight intelligent pupils, M. Leudet taking the management of the arm. At the first attempt, the head of the bone was dislodged from its place, and brought into the axilla; the second trial was followed by its complete reduction. An enormous swelling took place, almost immediately, beneath the pectoral muscles. The face became pale, and covered with sweat—the lips livid, and the pulsation in the radial artery ceased. A pulsating tumor formed in a few days in the axilla—gangrene of the limb succeeded—and, in a fortnight after the reduction the patient died.

*Dissection.* Hand and inside of the arm in a state of gangrene. The pectoralis major was almost completely torn across, and its fibres were separated by clots of blood. The upper portion of the short head of the biceps was ruptured also. All the muscles of the arm, shoulder, and outside of the chest, were infiltrated with blood. Between the pectoralis minor and latissimus dorsi, there was a large clot, on removing which, the axillary artery was found to be fairly torn across, a little above the origin of the subscapular. In order to discover the upper end of the vessel, it was necessary to dissect the subclavian, which was enlarged, as were the branches which arise from it. The axillary artery lay beneath the pectoralis minor, upon the rib, to which it adhered by means of coagulable lymph. This end of the vessel was narrowed, and the thoracic nerves flattened. The second rib was depressed, its periosteum slightly absorbed, and the bone itself a little rough. The head of the humerus was somewhat flattened at the part corresponding to the rib: the capsule was torn—the cartilage rough and ulcerated in parts. The inner margin of the glenoid cavity was fractured.

*Case* 2. Madame G. æt. 64, dislocated the head of the humerus into the axilla, by a fall, on Nov. 27th, 1823. Reduction was effected in the usual manner, by five assistants, seven weeks after the accident. The first attempt, which lasted seven or eight minutes, was unsuccessful; a second, and shorter one, effected the reduction, during which, the patient felt as though something "gave way" on the inside of the wrist. The patient was now found to be hemiplegiac on the right side. There was no motion, and sensation was very slight, especially in the arm. The eye of that side was half-closed, and there was a slight ecchymosis on the foot and lower third of the leg. M. Flaubert, considering the hemiplegia to be dependent on some extravasation within the head, produced by the agitation and violent efforts of the patient during the operation, bled her immediately. Purgatives were given, and rubefacients, with blisters, applied. The symptoms disappeared in part. In three months the leg was free from paralysis, but a degree of numbness and susceptibility to fatigue remained so late as February, 1826, the date of the last visit. The head of the humerus seemed to be drawn forwards, its motion was imperfect, the patient not being able to lift the hand to the mouth. The hand was useless, the thumb being extended, the other fingers semi-bent. The ring and little finger were quite insensible, and the heat of the limb appeared to be diminished.

*Case* 3. F., æt. 70, dislocated the head of the left humerus into the axilla, on Nov. 1st, 1825. Five weeks afterwards, M. Flaubert accomplished the reduction after two attempts. The patient immediately became affected with great constriction of the chest, a sense of suffocation, and the face became purple and injected. These symptoms were followed by an emphysematous effusion, stretching from under the clavicle, across the shoulder, to the middle of the back, where it gradually disappeared. The patient was pale, and the pulse weak, with nausea. In the left thigh and leg there was a sensation of cold and great numbness; the least touch upon the thigh caused exquisite pain. The patient was placed in bed, when she fell into a state of syncope for about an hour, on recovering from which, she complained of imperfection of vision, severe head-ache, and loss of motion in the right arm. Paralysis of the bladder, and of the upper and lower extremities of the left side, succeeded—difficulty of breathing followed after—sloughs formed upon the sacrum—and the patient died comatose on the 18th day after the reduction of the dislocation.

*Dissection*. The pectoralis major was bruised by the roller used for counter-extension, indeed, on the inside the fibres were reduced to a kind of reddish-brown bouillie. On the outer border of this muscle was a cavity containing bloody serum. All the nerves of the arm were united in a mass in the axilla by means of condensed cellular tissue, a circumstance apparently owing to the pressure produced by the head of the bone. On approaching the scaleni muscles, the four last nerves which go to form the axillary plexus, namely, the 6th, 7th, and 8th cervical, and 1st dorsal, were found torn from their origin in the spinal marrow! The brain and its membranes were sound. On opening the vertebral canal the dura mater was of a reddish brown. The tunica arachnoides was injected, particularly in the neck. The spinal marrow at this part presented a range of white spots, marking the place from which the roots of the nerves had been torn. The medulla at this point was thicker than natural, and of the consistence of a reddish-brown bouillie, the grey matter not being distinguishable from the white. The filaments were rather red and injected.

*Case* 4. A woman, æt. 45, dislocated the forearm backwards. No attempt at reduction was made till Dec. 9th, 1826, twenty-seven days after the accident, when it was attempted with the aid of seven pupils. Two such efforts were unsuccessful. The patient was then bled, and extension being made again, the ulna slipped into its place, whilst the radius which remained behind was easily pressed by the thumb into its situation. At the instant of reduction, a diminution *(étranglement)* of the elbow-joint took place, whilst a projection appeared above and below it. This was accompanied with a sound like that produced by the tearing of parts, and it seemed to those present, that the muscles which surround the articulation, with all the parts excepting the skin, were rent across. A considerable swelling followed, with a disposition to continual fits of syncope, whilst no pulsation could be felt in the radial artery. The pulse, however, returned next day, and the swelling gradually subsided. On the 26th December, she had an attack of pain in the right side of the chest, which was relieved by leeches. On the 4th Jannary she left the hospital, having tolerably free motion of the arm, none in the fore-arm, and but little mobility in the finger.

*Case* 5. The dislocation was that of the head of the humerus into the axilla, and had occurred fifteen days before M. Flaubert attempted its reduction. Before this could be effected, the operators were obliged to desist, in consequence of the patient's complaining of excessive pain in the wrist, with loss of motion in the hand and fore-arm, and numbness of the whole of the left lower extremity. Considerable constitutional irritation, and a severe attack of fever followed, whilst there took place, after a time, infiltration of the arm and fore-arm near the elbow, which lasted for several months. Subsequently, a slight power of flexion and extension were remarked in the fingers, but it did not increase. The fore-arm, at the time of the report, 1827, could be bent to a right angle with the arm, but there was much pain in the former, the neck, and the wrist; the whole limb, in fact, was wasted and useless.

*Case* 6. A man, æt. 40, received a dislocation of the left femur, the head of which rested a little above the great sciatic notch. Reduction was almost immediately effected by M. Flaubert. Two days after the accident the thigh became swollen, and the hip painful. The swelling extended to the knee, low fever was established, and on the fifth day after the operation the patient died.

*Dissection*. A large ecchymosis beneath the skin at the anterior and outer part of the thigh; rupture of the pyriformis, gemelli, and quadratus femoris muscles. The capsule torn, as was the ligamentum teres, close to the head of the femur. In the cavity of the joint there was a quantity of reddish pus, which communicated through the rent in the capsule with a depôt of bloody pus situated between the pectineus and adductor muscles. All the great organs were sound.

The perusal of the preceding cases will, we fear, give rise to opinions not highly favourable to the caution and judgment of M. Flaubert. An unlucky man might meet with one untoward case, but few surgeons of even the most vast experience have been visited with such a series of ugly accidents. In short, we conceive there can be no doubt that M. Flaubert used unwarrantable violence.

M. Dupuytren seems to participate in this opinion, though the judgment of the surgeon and the critic is varnished by a faint expression of regret at the bad fortune of M. Flaubert. Indeed the results of this gentleman's operations would seem to be cleverly and discreetly adduced as a foil to the more fortunate manipulations of the Baron. However this may be, a statement of the results of operations for reduction, in thirty-three cases of old dislocations, deserves the attention of the surgical reader.

Of the thirty-three cases twenty-five were instances of dislocation of the head of the humerus in various directions—five of dislocation of the femur—and three of that of the fore-arm.

Of the thirty-three dislocations—

| | | |
|---|---|---|
| 5 | were reduced from the | 5th to the 10th day. |
| 6 | .................... | 10th to the 20th. |
| 4 | .................... | 20th to the 30th. |
| 5 | .................... | 30th to the 40th. |
| 5 | .................... | 40th to the 50th. |
| 2 | .................... | 50th to the 60th. |
| 0 | .................... | 60th to the 70th. |
| 2 | .................... | 70th to the 80th. |
| 2 | .................... | 80th to the 90th. |
| 1 | .................... | 90th to the 100th. |
| 1 | .................... | at the end of 2 years.* |

The formidable number of twenty-six are drawn from the private and the public practice of M. Dupuytren. The dislocation of most brief duration had lasted for five days, the oldest had attained the age of eighty-two, and the majority had existed for twenty, forty, and fifty days.

One of the twenty-six patients died, in consequence of enormous laceration of the soft parts surrounding the articulation produced by the teeth of an enraged horse.

One patient suffered ever afterwards from difficulty in the motions of the hands and fingers. Reduction had been effected in this instance eight days after the reception of the accident.

In one child, ten years of age, dislocation of the elbow had existed for sixty-six days, and reduction was found to be impossible.

The remainder of the twenty-six patients of the Baron were quickly and perfectly cured.

The twenty-seventh and twenty-eighth cases are taken from Dessault. One of the dislocations (of the head of the humerus) was reduced on the forty-fifth day. Emphysema supervened upon the chest, but the patient, who was sixty years of age, had recovered on the thirtieth day after the operation. The second dislocation (of the humerus also) was reduced by Dessault on the ninetieth day. The patient was a female, aged 34; she was cured on the sixty-eighth day from the operation.

The twenty-ninth and thirtieth cases are taken from a work of Mothe, a surgeon to the Hôtel Dieu of Lyons. One dislocation had existed for seventeen days, and the other for five weeks prior to reduction.

The thirty-first case occurred to M. Sanson, second surgeon of the Hôtel Dieu of Paris. The patient was a female aged fifty-five, and the dislocation was reduced on the ninety-eighth day. The patient was soon well.

The thirty-second is extracted from a Treatise on Surgery by Delamotte, and is that of a physician named Desrosiers, who met with a dislocation of the humerus, which Delamotte reduced at the end of two months.

The thirty-third case is related in the fifth volume of the Memoirs of the Academy of Surgery, and displays so much of the incredible and inaccurate, as not to deserve more particular notice. The thigh is said to have been dislocated, and to have been reduced after the expiration of two years.

---

* A little research would extend this list from English sources. We are afraid of unseasonably augmenting the dimensions of this article.—*Eds.*

M. Dupuytren relates a case, possessing some features of interest. The reporters, indeed, who magnify at every turn the genius of their chief, assert that the fact is unequalled for singularity by any in the history of medicine. Our readers may judge for themselves.

*Case.* An old woman presented herself in the out-patient's room at the Hôtel Dieu, with a dislocation of the head of the humerus. The accident had happened six weeks previously, from a fall upon the hand with the arm extended forwards. The symptoms on her application were evidently those of dislocation downwards, into the axilla. But she stated that the symptoms had varied in the interval between the fall and her appearance at the Hôtel Dieu; and she said she had had the power of restoring the limb to its proper place, by efforts of her own and certain motions of the shoulder. When she had done this, she could move the limb, but still a certain degree of difficulty was experienced, and on making the attempt at any thing like an extensive motion, the symptoms of dislocation reappeared.

M. Dupuytren at first disregarded this story, and proceeded in the usual manner to reduce the limb. This appeared to have been done, and the patient declared that the limb was in place, as it had been before. On making a more particular examination of the shoulder, M. Dupuytren discovered that the deltoid was still more flattened than natural, and the prominence of the acromion still too great. Extension and counter-extension were resumed, and, after some slight efforts the head of the humerus was properly lodged in the glenoid cavity.

M. Dupuytren explains the circumstance with ingenious probability. The head of the humerus had undoubtedly been dislocated into the axilla, and had probably ruptured the capsular ligament. He supposes that the patient was enabled to replace it, partly on the anterior border of the glenoid fossa, partly in the contiguous subscapular fossa—that slight motions of the arm were compatible with the presence of the head of the bone in this situation—but, that more extensive movements occasioned its displacement.

These are the only observations of the Baron on dislocation of the head of the humerus, which appear to require any notice.

### ON CONGENITAL DISLOCATION OF THE FEMUR.

This is the subject of a clinical lecture of forty-eight pages in length. It does not follow the preceding article, in the published volume, but we introduce it here, for the purpose of connecting the remarks on dislocations. The substance of this lecture may be found in a memoir of M. Dupuytren's in the Repertoire d'Anatomie. That memoir we noticed in the year 1827; but as the abstract we then gave was brief, though sufficient to display the author's views, and as many of our present readers are probably unacquainted with it, we think we may be excused for its reintroduction upon this occasion. It would not be just to our readers nor to the lecturer to omit all notice of this, an interesting and important lecture, and we cannot present a more complete and at the same time condensed exposition of the lecturer's views, than the following short republication will afford.

It is well known that the most frequent kind of dislocation to which the hip-joint is liable is that of the head of the femur upwards and backwards on the dorsum of the ileum. It is also well known that of this dislocation there are two varieties—the first is the result of accident—the second is *consecutive*, the result of scrofulous ulceration in the joint. But M. Dupuytren in the memoir now before us, proposes to add to the list a third variety of this same dislocation, which, as it is found at birth, he terms " congenital."

The signs which characterise it, are shortening of the limb—presence of the head of the femur on the dorsum ilei—prominence of the trochanter major—retraction of almost all the muscles of the upper part of the thigh towards the crest of the ileum, where they form around the head of the femur a kind of cone, the base towards the os innominatum, the apex towards the trochanter—the almost entire denudation in consequence, of the tuber ischii—the rotation of the limb inwards—the obliquity of the thigh proportioned, of course, to the age and development of the pelvis—the meagreness of the limb, out of all proportion to the trunk and upper extremities, which are really well developed—and the imperfect motions, particularly of abduction and rotation. The upper part of the trunk of persons thus affected is thrown backwards, whilst the lumbar portion of the column projects as much forwards; the pelvis is placed almost horizontally on the femurs, and the ball of the feet alone touches the ground. In walking we observe them incline the body strongly towards the limb which is to support the weight, at which moment the head of the femur of that side is seen distinctly to rise on the dorsum ilei, in consequence of the superincumbent weight and sinking of the pelvis, and then they drag painfully forwards the opposite limb, the head of the femur of which is perceived not to rise but to sink, in consequence of its own weight drawing it down. This series of phenomena then is repeated each step the patient takes, and although locomotion to him is not so painful as it appears, still he is incapable of making any thing like a long journey.

In the recumbent posture, most of the symptoms of the dislocation in a great measure disappear, in consequence, no doubt, of the relaxation of the muscles and removal of the weight of the trunk. In this position of the body the surgeon can by a slight effort elongate the limb and shorten it again, that is, he can pull the head of the femur downwards or press it again upwards to the extent of two or even three inches, according to circumstances.

Let us look to the history of this complaint. Even at birth the prominence of the haunches, the obliquity of the femurs, &c. are perceptible, but in these cases the attention of the parents is seldom much directed to the malformation till the child begins to walk, and indeed even then its awkward efforts are attributed in general to weakness, &c. till the end of the third or fourth year, when the parent is at last convinced there must be something wrong. As the pelvis begins to be developed, (for it is a curious fact that the growth of the pelvis is never affected in these patients,) the symptoms which we have enumerated above become more marked, especially in females, and a person not acquainted with the true nature of the malady would consider it the consequence of scrophulous disease of the joint. But the previous history, the absence of all pain, swelling, abscess, fistula or cicatrix, and the simultaneous affection of both sides are sufficient to correct this error. At the same time it must be remarked that these individuals are for the most part of a lymphatic and scrophulous habit.

As the age of the person increases, and the superincumbent weight becomes of course greater, the heads of the femurs rise on the dorsum ilei till at last they almost touch the crista, the obliquity of the bones is increased, and the difficulty of motion proceeds at last so far as to incapacitate the patient from all active exercise.

As this of course is not a fatal disease, the opportunities for cultivating its pathology must be comparatively rare. In the cases which he has examined M. Dupuytren has found the acetabulum almost entirely obliterated or even entirely wanting; the head of the femur a little flattened on its internal and interior surface, and a sort of cotyloid cavity to lodge it, formed on the dorsum of the ileum, as happens in unreduced accidental dislocations. In one or two instances, he has seen the ligamentum teres elongated, and in some places worn apparently from the pressure and friction of the head of the femur.

The Baron puts some questions to himself and the reader as to the etiology. He asks, can the dislocation be consecutive to a disease which assailed the fœtus and disappeared before birth? Or can it be the result of an ante-natal accident? Or lastly, can it be owing to an original imperfection of the acetabulum, in other words, can it be a congenital malformation? He seems to incline to this position, but like a skilful disputant he has dealt his blows so equally to all his men of straw, that it is almost hard to say which is up or which is down.

M. Dupuytren makes some very sensible remarks on the treatment, which of course can be but palliative. As the weight of the trunk is the main agent in aggravating the displacement, repose is obviously indicated; but it is not necessary to confine patients to the recumbent posture; for in the act of sitting there is no stress on the femurs, the body resting entirely on the tuberosities of the ischia. Let these individuals then choose a profession which they can exercise when seated. Our author advises, likewise, the use of the cold bath, and the application of a bandage which encircles the pelvis, confines the trochanters, and keeps them of an uniform height, thus binding the ill-adapted parts together, and preventing that continual motion to which they are exposed. This practice, though it certainly will not cure the complaint, will give a great degree of support to the hip-joints, and prevent the progress of the displacement

In the course of eighteen years, M. Dupuytren has met with twenty cases of this kind, seventeen or eighteen of which have been females.

### On the Club-Foot.

This is a very frequent malformation. The Baron's observations upon it are brief, and are chiefly intended to urge practitioners to endeavour to remedy it early in life. There is no complaint, according to the Baron, in which a "stitch in time" is more necessary or more useful.

There appears to be three varieties of distorted, or club-foot. The most frequent is that to which the name of *varus* was anciently given. The point of the foot is directed inwards, and the sole is upturned in a similar direction, the patient walking on the outside of the foot, or sometimes even on a portion of its dorsum. The second variety, formerly called *valgus*, is much less frequent than the former ; in it, the foot is turned outwards. In the third form, the toes are turned absolutely backwards, and the whole of the foot is so reversed, that the patient walks completely on its dorsum.

The essential cause of all these malformations is luxation of one of the bones of the tarsus, the ligaments and muscles being only consecutively altered. The causes which favour or occasion these anomalies, during uterine existence, have attracted much attention ; but received no elucidation. In fact, we know nothing whatever about them. The external symptoms and the anatomical condition of the interior, have fully occupied anatomists and surgeons ; but none, observes the Baron, have accurately pointed out the changes that ensue in the affected limb.

The malformation may be limited to one foot, or occupy both. When the latter is the case, both limbs are usually equally developed, and nothing further need be said about them. When one foot is affected, the consequences are as follow.

In proportion as the individual grows older, the limb of the deformed side wastes. The cause is apparent; the patient instinctively employs the sound foot more than the diseased one, and the muscles and other tissues waste, or rather they do not sufficiently increase, because they are not proportionately used. Wasting of the muscles may, indeed, be remediable, but another and more serious effect is observed. The bones do not attain the same length on the affected, as on the unaffected side. As life advances, the difference is more marked. At birth, it is not present—at the age of ten years it has been very evident—at twenty, it is such as to set at defiance all remediable efforts. If the bones are shortened, the muscles and tendons must be shortened also. The tendo Achillis, at manhood, is so contracted as to render a high-heeled shoe indispensable, were the foot restored to its natural direction.

It is on these accounts, that the Baron impresses on surgeons and on parents, the paramount necessity of early attention to this ungainly malformation. Treatment in time is the only treatment. In infancy, the tissues are yielding and soft, and the foot can be placed in its natural relations without difficulty and without pain. As infancy recedes, the difficulties rise, and after a season become insurmountable.

And this, as we said before, is the sum of the lecture.

### On Laceration in the Centre of the Perinæum during Labour.

M. Dupuytren commences by observing, that rupture of the posterior commissure of the vulva, extending more or less into the perinæum, is a very frequent accident in labour. Sometimes the laceration is greater, involving the lower half of the posterior wall of the vagina, the whole extent of the perinæum, the sphincter of the anus, and the anterior wall of the rectum, to a certain height. But this is not the lesion that forms the subject of the present lecture; it is laceration of the central portion of the perinæum, without injury of the sphincter ani, or of the commissure of the vulva: through this laceration the fœtus is propelled.

It might, à priori, appear impossible for a body, of the bulk of a fœtus and its appurtenances, to pass through an opening confined to a space so limited as the perinæum of the female. What reason might reject, experience has confirmed. M. Dupuytren, or his reporters, adduce a long and an imposing list of names, as evidence in favour of the fact. Nédey, a surgeon of Besançon—Coutouly—Dr. Denman—Dr. Joubert—Meckel—Dr. J. Douglas—Moschener—Frank—Moreau, and others, are severally quoted, and their cases cited. For these details, interesting as they are in connexion with each other, we refer the curious reader to the lecture itself, and proceed to relate the particulars of a case which occurred in the wards of the Hôtel Dieu.

*Case.* Mad. B. æt. 38, was seized with the labour-pains of her first confinement in the morning of Sept. 3d, 1832. The child presented with the head in the first position, and labour went on well till the occiput appeared at the vulva, which was very narrow. Four hours after the commencement of the pains, two severe ones occurred, and the midwife felt a laceration of the perinæum take place under her hand, which was occupied in supporting it; at that instant, the head and the body of the fœtus issued at the aperture. The parts, it should be mentioned, were exposed in such a manner, that the midwife could distinctly see all that occurred. The child was born, and the cord and placenta were afterwards removed through the laceration. No hæmorrhage ensued.

Nothing was done until the tenth day after the confinement, when M. Guersent, jun. was consulted. He employed, in succession, lotions of the chloride of soda, the potassa fusa, the suture. For five days, the wound was united by the latter, excepting only a small fistulous point near the rectum. The suture was removed, and, in two days, the union was totally destroyed by some exertion of the patient.

On the 6th of October, the patient entered the Hôtel Dieu. The opening of the vulva was found to be situated remarkably forwards, a circumstance deserving of attention. Behind the vulva, and a little to the left, was another opening, irregularly rounded, and capable of admitting the extremities of the three fingers. The commissure of the vulva between them was perfect. Behind the lacerated opening was the anus, the sphincter of which was perfect also.

Such was the fact, and, after a few oratorical exclamations, M. Dupuytren goes to work in good earnest to explain, to comment, and to speculate. Persons in midwifery-practice, says he, will often have occasion to remark, that the vagina is placed very high and near the pubes, while the perinæum is proportionably lengthened. The vulva in these cases is contracted, although the vagina may present its natural dimensions. The diminution in size of the vulva results from the elongation of the perinæum, which latter may close a fourth, a third, or even so much as a half of the former. In females presenting this malformation, the finger, in exploring the vagina, must be carried downwards and backwards, instead of almost horizontally, and any instrument must be introduced in the same direction.

This state of the parts gives rise to several inconveniences. Sometimes it creates great difficulty in connexion—or the menses may be partially retained—or leucorrhœal matter may collect—or, in short, many other annoyances may ensue. The obstacles which it opposes to the process of parturition may be easily conceived. The head of the child, arrested by the narrow opening of the vulva, is directed against the perinæum. If the posterior commissure of the vulva is weak, it gives way—if resistant, the centre of the perinæum yields.

The malformation may either be congenital or accidental, that is, the consequence of cicatrization after burns, or wounds, or lacerations in previous confinements. M. Dupuytren digresses in a slight degree on the treatment of this encroaching perinæum. It must necessarily be divided, and appropriate means must be subsequently adopted to prevent improper cicatrization. In a pregnant female it is better to do this sufficiently early to allow of the formation of a firm cicatrix, prior to the supervention of labour. But circumstances may even render it advisable to divide the perinæum during that process. Dr. Champenois relates a case in which he performed the operation at this period, and prevented further laceration of the perinæum. The cause had been a burn in early life. Dr. Buet has related a curious, and rather a smutty case of accidental contraction of the vulva. A young lady managed to commit a faux pas, which ended in pregnancy—she concealed this, and supported alone a secret and most painful labour, terminating in extensive laceration of the labia and the perinæum. Union of the wound ensued, and occasioned such contraction that the little finger could scarcely be inserted in the vulva that was left. The young lady married, but the husband was unable to consummate the ceremony, on account of the obstinate virginity of his wife. Overjoyed at this proof of maiden chastity, he hastened to a surgeon to pick the lock. The man of art, intrusted by the lady with the sacred secret, divided the cicatrix, and prevented the re-establishment of the contraction by the introduction of lint.

M. Dupuytren observes that the position of the patient exercises an important influence on the presentation of the child. In the case related by Nédey, the woman was seated on the pot-de-chambre, when the birth and rupture of the perinæum occurred. In the instance related by M. Dupuytren himself, the mother was so propt up with pillows, as almost to assume the sitting posture. M. Dupuytren thinks, with M. Moreau, that too great a curvature backwards of the lower extremity of the sacrum and coccyx, or, what is tantamount to this, too decided a projection of the sacro-vertebral angle, must be ranked among the causes of the accident. Other causes may exist in the anatomical relations of the parts, but they have not been sufficiently nor accurately noted, and conjectures would be unsatisfactory and idle.

The last, and not the least important consideration is the treatment to be followed in these cases. The suture failed in the case received at the Hôtel Dieu, but M. Dupuytren feels well assured that it failed because it was withdrawn too soon. In all attempts to unite wounds of this description by the suture, the latter should be retained for a considerable length of time. A case is related in illustration of this maxim.

*Case.* Some years ago, M. Dupuytren was consulted by M. Gardien and another physician respecting the case of a young woman, who had been clandestinely confined. A complete rupture of the perinæum had occurred, and involved the lower inch of the anterior wall of the rectum. Several days had elapsed since the occurrence of the laceration. M. Dupuytren united the edges of the wound by the interrupted suture. In the course of a month, the girl was obliged to return to her parents, union not having yet taken place, and suppuration being still abundant. The threads of the suture had not cut through the skin, and M. Dupuytren recommended that they should not be removed until perfect union was obtained. Three or four years after this, a man and woman entered the private room of the Baron, to consult him on a circumstance embarrassing to both parties. The parties had been married, but the husband was unable to consummate the ceremony, in consequence of some difficulty at the entrance of the vagina. M. Dupuytren made an examination of the female, and found she was no other than his quondam patient. Union and subsequent contraction of the ruptured perinæum had occurred, and a firm cicatrix encroaching on the vagina had occasioned the ill-success of the husband. M. Dupuytren prescribed patience and perseverance, and these qualities were attended with their usual success. The female became pregnant, and was delivered without accident.

To return to the case received by M. Dupuytren in the Hôtel Dieu. We left the lady with a central opening in the perinæum, for which the suture had been unsuccessfully employed. M. Dupuytren directed her to recline continually upon her back, and the thighs were secured together by a bandage. The effect of this simple treatment was surprising. The opening contracted and finally closed, and on the 30th of November the patient left the hospital completely cured. The wound was closed, but probably some points yet remained ununited in the part of the vagina adjoining to the perinæum. The patient was enjoined to rest as much as possible, and to avoid connexion, in order that the cicatrization might be firm.

It will be observed by the reader of this lecture, that the Baron offers no novelty in treatment, and scarcely any in description. Yet the subject is deserving of the space we have devoted to it, and the case of laceration in the centre of the perinæum, is at least an addition to those already upon record.

### Fissures of the Anus.

We noticed this lecture in a late number of this Journal. We shall merely observe that the affection is not uncommon, particularly in the tenants of venereal hospitals. We have seen it in numerous instances at the Lock, and there seems some reason to believe that it frequently arises, like anal condylomata, from unnatural connexion.

Fissure of the anus consists in a long superficial ulceration, developed near the margin of the anus, in the radiated folds of the mucous membrane.

On separating the sides of the anus, and making the patient force down, a narrow chink is perceived; its surface red, its edges tumid and callous. The introduction of the finger is often necessary, to ascertain the extent to which it passes. It is situated more frequently on the lateral or posterior, than on the anterior face of the anus, and seldom attacks its whole circumference.

The severity of the affection depends on the spasmodic contraction of the sphincter, which occurs on the contact of the smallest foreign body.*

Division of the sphincter, and the application of the nitrate of silver, are the only remedies productive of advantage. M. Dupuytren, however, assures us, that spasm of the sphincter is the essence of the malady, and ulceration only a secondary phenomenon. He, therefore, aims at removing the former, in the expectation that the latter may then be speedily cured. With this view, he employs the following ointment.

℞. Axungiæ, p. s. vj.

Extracti belladonnæ,

Plumb. acet. āā p. m. j. M.

A tent of moderate size is smeared with this, and passed into the rectum, the size of the former being gradually increased. The constant employment of the ointment for some days frequently occasions complete relief.

For cases and details we refer to the Number of this Journal already mentioned. We cannot forbear from remarking, that the ulcer is more likely to constitute the cause of the spasm, than the spasm to occasion, or even essentially to maintain the ulcer. We have lately tried the Baron's method in these cases. We regret to state that it did not succeed. We found that a single touch of the nitrate of silver did more to allay the spasmodic contraction of the sphincter than any description of sedative or narcotic.

### Some Remarks on the Employment of Cauteries and Moxas.

These are erected into a separate clinical article. They may be readily compressed into a very narrow compass. We need scarcely observe, with the accuracy of the Baron, that they occasion pain and produce an eschar, consequences equally obvious and familiar. We shall glance at the situations in which issues are most convenient, and extract a practical remark of the lecturer on the best mode of keeping them open.

The best part in the arm for the establishment of an issue is the slight depression situated at the point of insertion of the deltoid. In the thigh, the most eligible spot is a few fingers' breadth above the internal condyle of the femur, over the cellular partition between the quadriceps extensor and the adductors. In the leg, we may select the interval between the internal border of the tibia and the gastrocnemius.

M. Dupuytren recommends, with questionable judgment, that the issue

---

* No. 38, p. 542-3.

should be slowly made. The suffering of the patient prompts, and almost compels, the humane operator, to accelerate the work of suffering.

There are two modes of keeping issues open. One is to apply and to retain some foreign substance, as a bean, or a piece of lint; the other consists in the frequent application of the caustic. Mr. Brodie has directed the attention of surgeons to the superiority of the latter method. M. Dupuytren animadverts, like Mr. Brodie, on the disadvantages attendant on the former—on its want of efficacy upon one hand, and the irritation of the system which it sometimes excites upon the other. It is more particularly for the reason last mentioned, that M. Dupuytren condemns this method. But his plan is not identical with that of Mr. Brodie. The latter gentleman keeps the issue *open* by occasional applications of the caustic; the former recommends a succession of issues, allowing one to close before he repeats the cauterizing process. Mr. Brodie's method has these advantages; it is productive of less pain, because the caustic is more lightly used; and probably its efficiency is greater, because the issue is kept constantly discharging to a certain point.

### Abscess in the Right Iliac Fossa.

This, like other articles in the volumes before us, has been noticed in former Numbers of this Journal. Abscess, occurring in the right iliac fossa, has been noticed upon more than one occasion.* We will not omit all notice of the affection, but content ourselves with offering a sketch of the more important facts connected with its history and treatment.

The more frequent occurrence of inflammatory swellings in the right iliac fossa than in the left, would appear to be explained in a sufficient degree by their respective anatomical relations. The cæcum unites with the small intestines in a manner so abrupt, that a remarkable contraction, the ileo-cæcal valve is presented. The food become fæces, has here to ascend against the laws of gravity, and probably it is owing to the combination of these circumstances :—The valve, the cæcal cul-de-sac, and the ascending course which the excrement must take, that lodgements of foreign bodies, such as fish-bones, fruit-stones, and so forth, is frequently observed in this part of the alimentary canal. The cæcum is invested only in front by the peritoneum; behind, it is implanted in a mass of loose cellular tissue, which forms its cushion in the iliac fossa, and connects it with the iliac muscle. Such are the anatomical conditions displayed by the cæcum and commencing portion of the ascending colon. On the left side, the arrangement of the colon is different. Its sigmoid flexure is almost surrounded by peritoneum—no contraction of calibre is remarked—the passage of the excrement is promoted by its descent.

It would seem that precursory symptoms are usually experienced prior to the formation, or, at least, to the appearance of the abscess. The patient

---

* *Vide* No. 19, for January, 1829—p. 217-221.—Eds.

may have committed some imprudences in regimen, and may have suffered from some irregularity of bowels. Constipation, or diarrhœa, or colic pains in the right iliac fossa, may have been remarked for some days or some months, and vomiting may have occurred in connexion with those symptoms. The premonitory symptoms, in short, are vague, and such symptoms occur upon many occasions without any abscess supervening.

Predisposing causes are various and vague. Youth would appear more prone to the disease than advanced or even than mature life; for of sixteen cases carefully collected, eleven were in individuals under thirty years of age. Males are more subject to the malady than females. Autumn and the end of Summer are the seasons in which it is most rife. The two latter circumstances probably are due to the greater excesses and more violent exertion of men than women. Such is not an unlikely explanation, though it does not occur to M. Dupuytren. That this explanation is consistent with the facts will appear when we state that persons in laborious and unhealthy occupations are those most frequently the subjects of the disease. House-painters, colour-grinders, copper-turners, are the artisans enumerated as especial sufferers by the lecturer. Those occupied with sedentary or with literary pursuits have been affected, after labouring under great derangement of the organs of digestion. Peasants or workmen leaving the country to reside in Paris have, in many instances, laboured under this complaint, a circumstance attributed by M. Dupuytren to the miserable fare of the Parisian *ouvrier*, especially in the fine season when employment is scarce.

The symptoms of the disease, when actually established, are not very equivocal. A fixed pain is felt in the iliac region, and circumscribed swelling is probably apparent. On tapping or on pressing the affected part, it is found more tense and resistant than natural, and a tumor may be distinguished, of various volume, of considerable hardness, and displaying more tenderness than any other part of the abdomen, and appearing as if it reposed upon the cæcum. The patient complains of constipation, of colic, and of inability to expel the intestinal flatus by the rectum. Sometimes there is fever; usually the general symptoms are slight.

If the case proceeds, as it usually does, to a favourable termination, the tumor slowly disappears; deep hardness remaining for a lengthened period to indicate the seat of the disease. Of sixteen cases collected and compared by M. Meinière, eleven terminated in this successful manner.

Less fortunate cases do, however, occur. Pulsating pain is experienced in the tumor, which increases in dimensions, becomes softer, fluctuates, and bursts. Happily the abscess opens in many instances into the intestine; when a pressing desire is felt to go to stool, and purulent evacuations from the bowels ensue, accompanied with a diminution of the volume of the tumor. Recovery in such cases is generally rapid. Sometimes the abscess bursts into the bladder as well as the intestine—sometimes it is discharged into the vagina—sometimes it opens on the surface of the body. This latter termination is almost always fatal, and chiefly, perhaps, upon this account:—that the bottom of the abscess is the most dependent part in the usual position on the back, and the matter is consequently prevented from obtaining a free and ready exit. The results are of course the formation and extension of fresh purulent collections, the introduction of air, and confinement of putrid and noxious discharge.

A curious circumstance is noticed. Purulent matter passes from the abscess into the intestine, and fæces escape from the latter to the abscess. The constant pressure of the abdominal parietes is probably the most efficient agent in producing this exchange of contents.

Sometimes inflammation extends to the peritoneum, or cellular tissue seated behind it. This is a danger to be borne in mind, and calculated to inspire uneasiness and caution in cases which wear the most favourable aspect.

The Baron makes some remarks on the affections with which the present might perhaps be confounded. 1. Inflammatory swellings are sometimes developed in the right or the left iliac fossa, dependent upon inflammation of the iliac fascia. 2. It is not unfrequent after confinement to observe a swelling in the iliac fossa, originating in the round or the broad ligaments of the uterus, and extending to the neighbouring cellular tissue and the iliac fossa. Sometimes such abscesses are discharged into the womb; sometimes they burst into the vagina. 3. The iliac fossæ may become the seat of collections of matter, arising from caries of the neighbouring bones or disease of the contiguous ligaments. This species of collection is soft and fluctuates as soon as it appears, a sufficiently distinctive feature. M. Dupuytren observes, in concluding his notice of these abscesses resembling the one we are considering, that he frequently has witnessed very gross mistakes in the diagnosis of the latter.

The prognosis is not in general unfavourable. Of sixteen patients affected with abscess in the right iliac fossa, one, and one only died. The supervention of peritonitis is usually fatal. The treatment is simple, obvious, and efficient. Local and general bleeding, fomentations, poultices, baths, lavements, and laxative drinks, are the artillery of M. Dupuytren. The name of calomel, that bug-bear of French physic, is not breathed, and the surgeon of the Hôtel Dieu does not hesitate to use the lancet, but dreads to prescribe the pill. In this country calomel with mild aperients would form an important item in the remedies.

For cases illustrative of the description and remarks we must refer to other papers in this Journal.*

Here we must bring this article to a conclusion. In our next we shall resume the review of the third, and enter on that of the fourth volume of these lectures, and we trust we shall present in a concise, yet sufficiently ample, form, the substance of what is really a valuable work.

---

* Several are contained in the Number previously alluded to—that for January, 1829.—Eds.

